# Closed‐Loop Radiative Cooling Mulch Upcycled From Agricultural Residues for Efficient Soil Heat–Water Stress Mitigation

**DOI:** 10.1002/advs.75987

**Published:** 2026-06-09

**Authors:** Hao Li, Dong Lv, Yang Fu, Cancheng Jiang, Wenqi Wang, Ze Li, Lin Liang, Jiayu Du, Jie Tan, Yihao Zhu, Wenjie Liu, Lamfeddal Kouisni, Kaixin Lin, Chi Yan Tso

**Affiliations:** ^1^ School of Energy and Environment City University of Hong Kong Hong Kong SAR China; ^2^ School of Energy and Environmental Engineering University of Science and Technology Beijing Beijing China; ^3^ Shunde Innovation School University of Science and Technology Beijing Foshan China; ^4^ African Sustainable Agriculture Research Institute (ASARI) College of Agriculture and Environmental Science University Mohammed VI Polytechnic (UM6P) Laâyoune Morocco

**Keywords:** agricultural waste recycling, passive radiative cooling, soil thermal and moisture management, sustainable agricultural development

## Abstract

Global warming is intensifying coupled heat–water stress in agriculture, necessitating scalable strategies for cooling and moisture retention. Passive radiative cooling (PRC) presents a promising avenue, yet current PRC films predominantly rely on expensive non‐agricultural feedstocks and involve energy‐ or solvent‐intensive manufacturing processes, while limited end‐of‐life environmental compatibility hampers alignment with agricultural systems. Herein, we present a sustainable radiative cooling mulch (SRCM) derived from waste maize leaves, engineered to establish an “agricultural residue–material–soil” closed loop. Through spontaneous hydrogen‐bond‐mediated self‐assembly requiring no external energy, we engineered a hierarchically porous, self‐reinforced fibrous network that combines application‐grade mechanical robustness (tensile strength up to 17.8 MPa) with a high solar reflectance of 93.3% and a mid‐infrared emissivity of 92.6%. In outdoor tests, relative to bare soil, SRCM lowers soil temperature by up to 18°C and suppresses evaporative water loss by up to 85.8%, markedly improving early‐stage bok choy growth with 5.3‐fold higher germination and 220% greater biomass. Furthermore, SRCM demonstrates excellent recyclability via aqueous reprocessing and exhibits complete biodegradability and biosafety at the end of its life cycle. This work offers a scalable, closed‐loop strategy at the heat–water–food–energy nexus, advancing sustainable circular agriculture.

## Graphical abstract

Conventional passive radiative cooling films rely on costly feedstocks and energy‐intensive fabrication, with poor end‐of‐life compatibility in agriculture. To address this challenge, a sustainable radiative cooling mulch (SRCM) is developed from waste maize leaves via spontaneous hydrogen‐bond self‐assembly. The SRCM exhibits high solar reflectance, strong mid‐infrared emissivity, and full biodegradability, showing great promise in cooling, moisture retention, and circular agriculture.

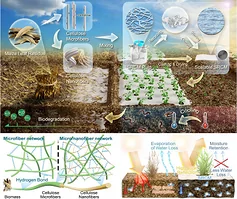

## 1 Introduction

Agriculture, the cornerstone of human survival, is highly dependent on suitable climate and water resources for stability and productivity [1, 2]. These foundations face growing threats from extreme heatwaves and droughts driven by ongoing global warming [3, 4, 5]. Quantitative global assessments indicate that each 1°C increase in global mean temperature leads to average yield losses of 3.1%–7.4% for major staple crops, highlighting the real‐world urgency of developing scalable strategies to protect agricultural productivity under intensifying climate stress [6]. Conventional measures such as greenhouses help mitigate these stresses, but they mainly regulate the aboveground microclimate [7, 8]. In contrast, the root zone, which is critical for crop growth and controls water and nutrient acquisition, has received much less attention. This gap is especially important during early growth in hot climates when the canopy is still sparse and provides little shade. Direct solar loading on bare soil increases subsurface heat flux and raises root zone temperature. Once thermal thresholds are crossed, root vitality declines and water and nutrient uptake are impaired, which in turn leads to substantial losses in yield and quality [9, 10, 11]. At the same time, solar‐induced soil warming accelerates evaporative water loss, thereby exacerbating agricultural water stress [12]. Therefore, regulating the root‐zone thermal condition is crucial to ensuring optimal plant growth.

Modern cooling techniques (e.g., refrigeration and ventilation systems) for plant cultivation have advanced rapidly in recent decades, yet they involve unavoidable trade‐offs among energy consumption, water use, and operation costs [13, 14, 15, 16]. By contrast, mulches offer a low‐cost, passive alternative that can suppress evaporation, conserve soil moisture, and control weeds, thereby enabling soil heat and mass management without additional energy or water input. However, their optical properties are generally not optimized for thermal regulation. For transparent mulches, high transmittance in the solar spectrum allows substantial solar radiation to reach the soil surface, causing soil heating and heat accumulation via a greenhouse effect. For opaque mulches designed to block sunlight, both solar‐absorbing (e.g., black) and solar‐reflective (e.g., silver‐white) types face inherent thermal limitations. Absorbing mulches readily convert incident sunlight into heat, leading to substantial warming. Reflective mulches are used to mitigate this effect, but their performance is fundamentally constrained by limited solar reflectivity, which for common commercial products typically remains below 60% [13, 17]. As a result, the remaining absorbed solar energy leads to parasitic heat gain within the mulch layer. This accumulated heat is then conducted downward into the soil, partially offsetting any cooling benefit and still contributing to undesirable soil warming. In addition, the widespread use of plastic mulches increases labor and recovery costs and poses a serious risk of microplastic pollution in agricultural soils and surrounding environments [18, 19, 20].

By reflecting solar radiation and emitting heat through the atmospheric window, passive radiative cooling (PRC) provides a promising route to zero‐energy cooling mulches [21, 22]. However, the deployment of current PRC technologies in agriculture remains limited (Table S1). Photonic micro/nanostructures, for example, are hindered by their high cost, sensitivity to contamination and mechanical damage, and difficulties in large‐scale manufacturing [23, 24]. Polymer‐based composites have therefore attracted attention for their potential advantages in cost and scalability. Yet conventional inorganic‐filled porous polymers typically require either very high filler loadings or energy‐intensive processing to achieve high solar reflectivity [25, 26]. While phase‐separation‐derived porous polymer films circumvent inorganic fillers, they are typically plagued by toxic‐solvent dependence and persistent non‐degradability [27], together undermining both their cost‐effectiveness and environmental benefits. In response, biopolymer‐based PRC materials have been widely explored. Nonetheless, attaining the desired optical properties often relies on chemical solvents, complex spinning, or high‐pressure physical foaming techniques, which increase environmental burden or energy consumption [28, 29, 30]. Furthermore, some biopolymers exhibit insufficient degradation rates [31], leading to incompatibility with crop rotation schedules and leaving persistent residues that inhibit water and nutrient uptake by succeeding crops. Cellulose, by contrast, is abundant, nontoxic, biodegradable, and intrinsically emissive in the infrared, making it an attractive feedstock for sustainable radiative cooling. Representative cellulose‐based PRC systems, including cooling wood [32, 33], aerogels [34, 35], fabrics [36, 37, 38], and coatings [39], have been widely explored for buildings, personal thermal management, and energy harvesting, yet their agricultural translation remains limited by persistent trade‐offs in form factor, manufacturability, scalability, cost, and environmental sustainability (Table S2). Even the few recent cellulose‐based systems reported for agricultural thermal management have not resolved this mismatch [17, 40]. More broadly, the forestry‐derived feedstocks used in most cellulose‐based PRC systems are misaligned with agriculture's need for locally sourced, low‐carbon raw materials [41]. Therefore, it is crucial to develop an agricultural PRC mulch that is specifically tailored to reconcile high cooling performance with cost‐effectiveness and sustainability, thereby overcoming current technological bottlenecks.

Agricultural waste residues are the nonedible fractions left in fields after harvest, such as leaves and straw, constituting an immense biomass resource, with an annual production exceeding 730 million tons in China alone [42, 43]. However, prevailing disposal practices, including open‐field burning and improper dumping, impose severe environmental burdens [44]. Critically, these residues consist predominantly of cellulose, a dielectric material with a large electronic band gap that makes it intrinsically non‐absorptive across the visible spectrum [45]. Concurrently, characteristic molecular vibrations in cellulose give rise to strong thermal emissivity within the 8–13 µm atmospheric window [46]. This combination of properties suggests that in situ valorization of agricultural residues into mulching materials offers a compelling route to address the current shortage of agriculture‐specific PRC mulches. However, translating this potential into practical PRC mulches demands a scalable, low‐footprint manufacturing route that converts heterogeneous residues into optically optimized, mechanically robust mulches while minimizing energy demand and chemical inputs and ensuring end‐of‐life environmental compatibility [47, 48, 49, 50, 51].

**FIGURE 1:**
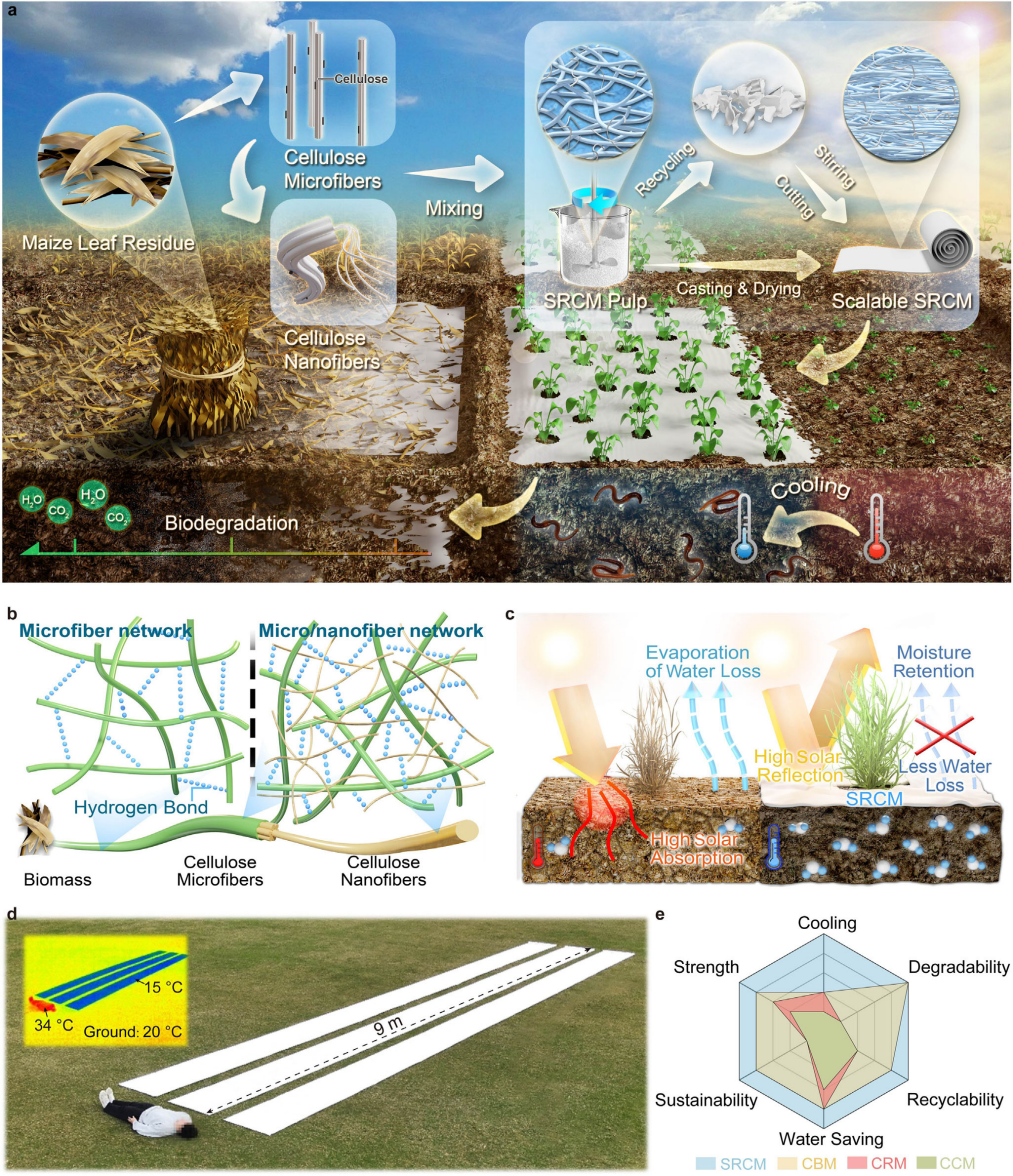
Design principle and visual appearance of SRCM. (a) The closed‐loop life cycle concept for SRCM. (b) Enhancement of SRCM's mechanical properties via an in situ self‐reinforcement strategy. (c) Hydrothermal management of the soil by using SRCMs. (d) Photograph of large‐scale SRCMs (9 m long) on grassland; the inset shows an infrared thermal image indicating cooling performance. (e) Radar chart comparing the performance of SRCM with commercial biodegradable (CBM), reflective (CRM), and conventional (CCM) mulches.

Here, we propose a closed‐loop strategy conceptualized as “originating from agriculture, serving agriculture, and returning to agriculture” (Figure 1a). In this approach, agricultural biomass is used exclusively for mulch fabrication, applied in the field to support crop growth, and then allowed to naturally degrade after use, thereby re‐entering the soil carbon cycle and reintegrating biomass into the agroecosystem. We developed a scalable, all‐biomass sustainable radiative cooling mulch (SRCM) derived from abundant waste maize leaves. This material combines the mechanical toughness required for the field application with excellent PRC performance, enabling stable daytime sub‐ambient cooling. Outdoor plantation experiments show that SRCM markedly improves crops’ early‐stage growth and development, achieving a germination rate of 95% and increasing seedling biomass by 49% compared with commercial reflective mulches, and by approximately threefold relative to bare soil. In terms of environmental compatibility, SRCM exhibits a multifaceted sustainability profile. It undergoes complete natural degradation within three months without any detectable ecotoxicity and maintains benign interactions with soil biota, indicating good compatibility with soil ecosystems and subsequent planting cycles, while contributing to reduced greenhouse gas emissions under controlled conditions. Accordingly, SRCM offers an efficient, scalable, and closed‐loop sustainable solution for root‐zone thermal and moisture management, helping to mitigate compound heat‐drought stress, promote crop production, and advance circular agriculture.

## 2 Results and Discussion

### 2.1 Design and Working Mechanism of SRCM

In nature, the sophisticated architecture and outstanding mechanical properties of many biological materials continually inspire the development of high‐performance artificial systems [52, 53, 54]. For example, the remarkable strength of spider silk arises from the synergistic interaction between its crystalline domains and amorphous matrix [55], while the structural integrity of plant cell walls depends on the intricate interlocking of nanofibrils with the surrounding matrix [56]. Inspired by these principles, we engineered a mechanically self‐reinforcing, hierarchical porous structure in which cellulose nanofibrils (CNFs) act as a nanoscale “mortar” to bind homologous cellulose microfibers (CMFs). Both cellulosic components are extracted from abundant, low‐value maize leaves [57], and co‐assembled via a simple, scalable aqueous casting process that avoids toxic reagents as well as energy‐intensive extrusion or hot‐pressing (Figure 1a and Figure S1). This bio‐inspired co‐assembly spontaneously forms a densely interconnected, hierarchical network that imparts field‐ready mechanical robustness, far exceeding that of homogeneous microfiber systems (Figure 1b). Simultaneously, the highly porous architecture affords strong solar reflectivity, and the intrinsically high thermal emissivity of cellulose together enables effective PRC performance. As a result, SRCM can regulate soil thermal‐moisture conditions by mitigating excessive daytime heating and reducing evaporative water loss, thereby creating a more stable and favorable root‐zone environment for vigorous crop growth (Figure 1c). The evaporation‐driven fabrication process can be adapted for large‐scale production in practical applications (Figure 1d). Comprehensive benchmarking reveals that SRCM outperforms commercial mulches in mechanical strength, cooling performance, water saving, degradability, and recyclability (Figure 1e and Table S3). These superior properties, combined with eco‐friendly manufacturing, position SRCM as an ideal next‐generation sustainable solution for agriculture in hot climates.

### 2.2 Fabrication of SRCM and Enhancement of Mechanical Performance

**FIGURE 2:**
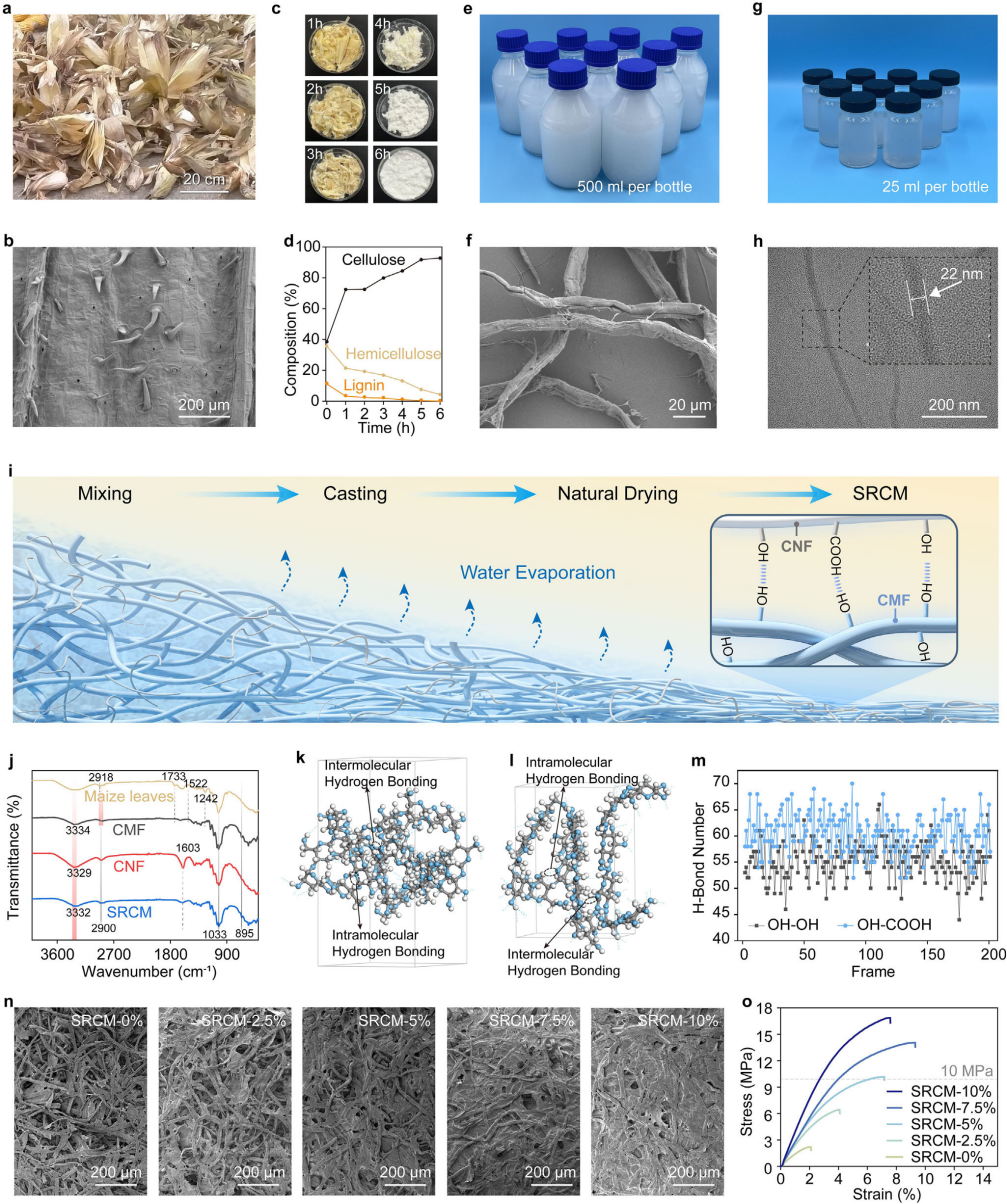
Fabrication process, multiscale structural characterization, and mechanical performance enhancement of SRCM. (a) Discarded maize leaves as feedstock. (b) Multiscale surface morphology of raw leaves. (c) Visual transition after sequential delignification. (d) Time‑resolved compositional evolution to high‑purity cellulose. (e, f) Homogenization‐derived CMF suspensions comprising shortened micro‐scale fibers. (g, h) TEMPO‐oxidized CNF dispersions comprising high‐aspect ratio nanofibers. (i) Schematic illustrating castable CMF/CNF assembly into SRCM during water evaporation. (j) FTIR spectra of Maize leaves, CMF, CNF, and SRCM. (k) Atomistic configuration of the CMF–CMF interface. (l) Atomistic configuration of the CMF–CNF interface. (m) Statistical distribution of distinct hydrogen bonding interactions. Morphological densification (n) and tensile strength (o) as functions of CNF content.

The fabrication strategy of SRCM is centered on converting single, low‐value feedstock, discarded maize leaves, into two structurally distinct cellulosic components for co‐assembly. The maize leaves (Figure 2a) feature a multiscale surface of dense lamellae and coarse fiber bundles with spiny protrusions, wrinkled grooves, and stomata, while their cellulose fibers remain embedded in the native matrix (Figure 2b). The process begins with sequential delignification, which selectively removes lignin and hemicellulose from the native matrix (Figure 2c,d and Figure S2). This treatment is accompanied by a visible color change from yellowish‐brown to white and an increase in the relative cellulose content from approximately 40%–92%. FTIR spectroscopy confirms the purification efficacy (Figure 2j), with the bleached pulp exhibiting complete disappearance of the ester peak at 1730 cm^−^^1^ and significant attenuation of lignin characteristic peaks at 1515 and 1240 cm^−^^1^. The C─H stretching vibration undergoes a 20 cm^−^^1^ red shift to 2900 cm^−^^1^, indicating the removal of lignin side chains and hemicellulose acetyl groups. The purified pulp is subsequently homogenized to yield CMFs with diameters of 10–20 µm (Figure 2e,f). XRD patterns reveal sharper diffraction peaks compared to the raw leaves, demonstrating that CMF retains the highly crystalline cellulose I framework (Figure S3). A portion of these CMFs is then further processed via TEMPO‐mediated oxidation to produce filamentous, high‐aspect ratio CNFs with sub‐22 nm diameters (Figure 2g,h). Finally, SRCM is obtained through a simple casting process, in which water evaporation from a CMF slurry containing a small fraction of CNFs drives co‐assembly of the components into a dense, cohesive structure (Figure 2i).

The resulting SRCM exhibits distinctive intermolecular interactions and structural characteristics. FTIR analysis (Figure 2j) reveals that TEMPO‐oxidized CNF displays a prominent carboxylate (─COO^−^) characteristic peak at 1603 cm^−^^1^ compared to CMF, indicating selective functionalization of C6 hydroxyl groups. The retention of this characteristic peak in the SRCM composite film confirms the successful incorporation of CNFs. Meanwhile, all samples retain the C─H stretching vibration peak at 2900 cm^−^^1^, the C─O─C glycosidic bond peak at 1033 cm^−^^1^, and the β‐1,4‐glycosidic bond peak at 895 cm^−^^1^, confirming that the cellulose backbone structure remains intact throughout the fabrication process. Compared to pure CMF, the hydroxyl O–H stretching vibration peak in SRCM undergoes a slight red shift from 3334 to 3332 cm^−^^1^, indicating changes in the hydrogen bonding environment associated with hydrogen bond interactions between micro‐ and nanocellulose fibers. X‐ray diffraction (XRD) patterns corroborated the structural integrity of SRCM, which retained the characteristic cellulose I peaks at 14.6°, 16.6°, and 22.6°, confirming that co‐assembly induced no crystal phase transition (Figure S3). To elucidate the mechanism underlying the enhanced interfacial interactions, molecular dynamics (MD) simulations were performed. Both the MD models of CMF–CMF interface (Figure 2k) and the CMF–CNF interface (Figure 2l) showed the existence of intermolecular bonding and intramolecular bonding. While both systems exhibited similar hydrogen bond length distributions ranging from 1.7 to 1.8 Å (Figure S4), the introduction of carboxyl groups fundamentally altered the bonding network topology. Compared to the unmodified system, the ─COOH groups induced a proliferation of total hydrogen bonds (Figure 2m), driven primarily by a surge in intermolecular bonding density alongside robust intramolecular bonding (Figure S5).

To translate these strong molecular‐level interactions into macroscopic performance, we tailored the material morphology and mechanics by adjusting the CMF/CNF mass ratio (SRCM‐X%). Pure CMF (SRCM‐0%) self‐assembled into a loose, porous network due to limited physical entanglement, resulting in poor mechanical stability. The incorporation of CNF, however, acted as a “nano‐binder,” bridging adjacent microfibers to enhance interlayer adhesion and reduce porosity (Figure S6). When the CNF content exceeds 5%, the interpenetrating multi‐scale fiber structure significantly improved network continuity and compactness (Figure 2n). Consistent with the interfacial strengthening predicted by MD simulations, uniaxial tensile tests revealed a monotonic increase in tensile strength with rising CNF content (Figure S7 and Figure 2o). SRCM‐5% reaches a tensile strength above 10 MPa, exceeding typical commercial plastic mulch requirements [58]. Further increasing the CNF loading raises the ultimate strength to 17.8 MPa, representing an eightfold improvement over SRCM‐0%. This enhancement arises from the high specific surface area of CNFs and their abundant hydrogen‐bonding sites, which promote efficient stress transfer across the CMF–CNF interface and effectively inhibit crack propagation.

### 2.3 Optical Properties of SRCM

**FIGURE 3:**
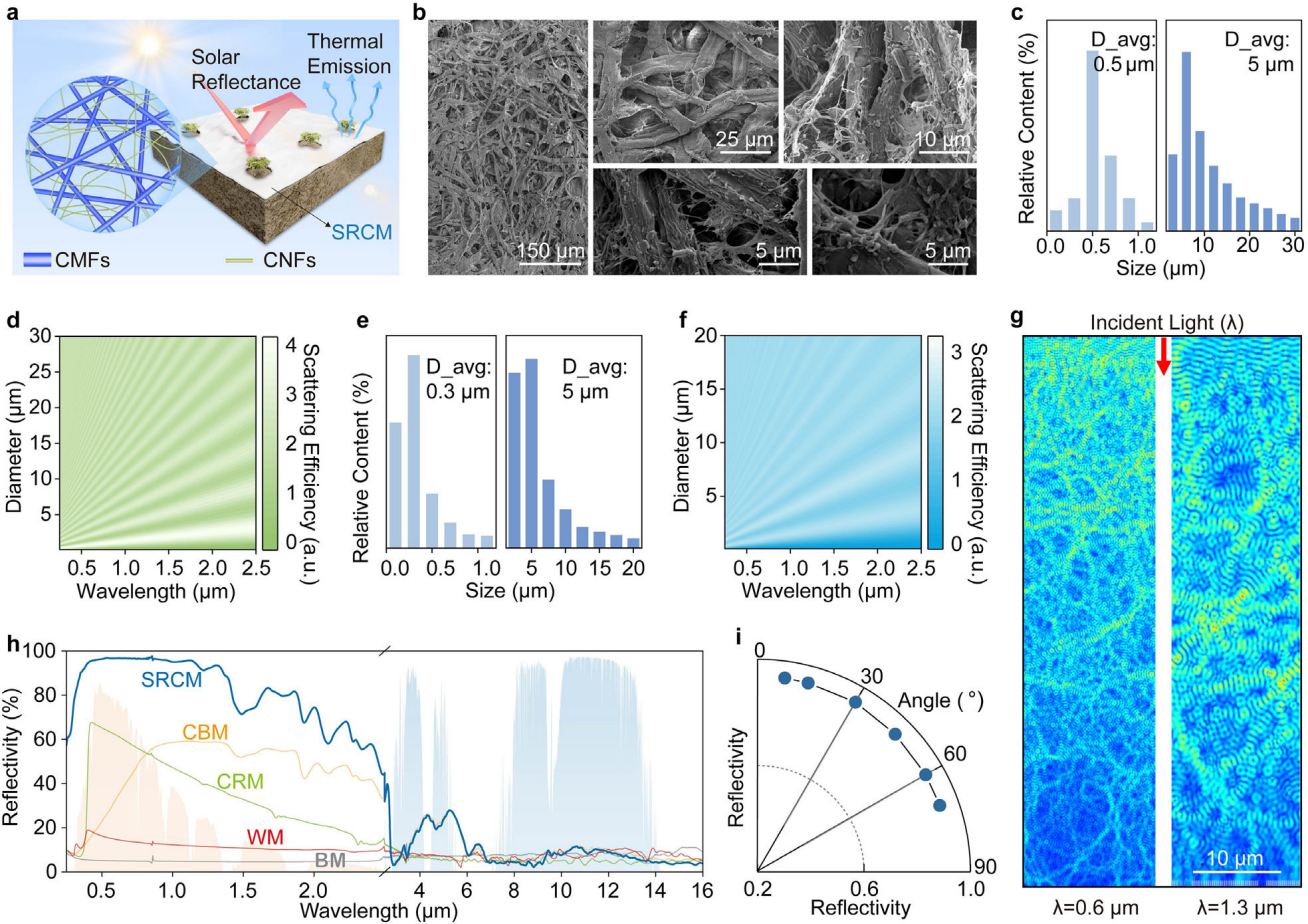
Optical properties of SRCM. (a) Schematic illustration of SRCM for soil thermal management via radiative cooling. (b) Microstructure of SRCM‐5%. (c) Size distributions of nanofibers (average diameter: 0.5 µm) and microfibers (average diameter: 5 µm) in SRCM. (d) Scattering efficiency of cellulose fibers with various diameters. (e) Size distributions of nanopores (average size: 0.3 µm) and micropores (average size: 5 µm) in SRCM. (f) Scattering efficiency of pores with various sizes. (g) Electromagnetic simulations of the electric field distribution in SRCM under illumination at wavelengths of 0.6 and 1.3 µm. (h) Measured spectral properties of SRCM, a commercial biodegradable mulch (CBM), a commercial reflective mulch (CRM), a black mulch (BM), and a white mulch (WM) over the 0.25–16 µm wavelength range. The orange shaded region indicates the normalized solar spectral irradiance, and the blue shaded region denotes the atmospheric longwave transmittance window. (i) Angular reflectivity value of SRCM.

The cooling capacity of a PRC material is governed by its net radiative energy balance (Figure S8), which requires high solar reflectivity in the 0.25–2.5 µm range to minimize solar heat gain and high thermal emissivity within the 8–13 µm atmospheric window to maximize radiative heat loss (Figure 3a). SRCM satisfies both criteria through the interplay of intrinsic material properties and engineered structure. Its high thermal emissivity arises from the cellulose backbone, whereas its high solar reflectivity is structural in origin. SEM imaging reveals a multiscale architecture in which CNFs permeate the interstices of CMFs scaffolds (Figure 3b). This rationally designed co‐assembly produces bimodal distributions in both fiber diameters (peaking at ∼0.5 and ∼5 µm, Figure 3c) and pore sizes (peaking at ∼0.3 and ∼5 µm, Figure 3e). To elucidate the origin of the high solar reflectivity, we conducted numerical simulations based on the Mie theory. The results show that light scattering is distributed across the structural hierarchy. Microscale features, including CMFs and larger pores, dominate scattering in the near‐infrared region. In contrast, submicron features, such as CNFs and smaller pores, efficiently scatter visible and ultraviolet light (Figure 3d,f, and Figures S9) and S10. The broadband solar reflectivity of SRCM therefore arises from the combined contribution of these multiscale scattering mechanisms. To further assess the robustness of this idealized size‐resolved interpretation, supplementary sensitivity calculations were performed by varying feature aspect ratio and local inter‐feature spacing (Figures S11) and S12. These results show that moderate shape anisotropy and local coupling mainly modify the scattering strength quantitatively, while preserving the principal wavelength‐dependent trend governed by the multiscale feature sizes.

To relate the multiscale architecture to the macroscopic optical response, we simulated the electric‐field distribution within an experimentally characterized SRCM cross‐section (Figure 3g and Figures S13) and S14. The simulations reveal a pronounced wavelength‐dependent penetration depth. Longer wavelengths in the 1000–1300 nm range penetrate more deeply into the structure, whereas shorter wavelengths in the 400–600 nm range are efficiently scattered near the surface. This wavelength‐selective behavior underpins the experimentally measured spectral response (Figure 3h). Consistent with the simulations, SRCM exhibits highly efficient visible‐light scattering, with a reflectivity of 96.4%. Reflectivity in the near‐infrared is slightly lower because of greater penetration depth yet remains high at 90.1%. Together with 90.1% ultraviolet reflectivity, these contributions yield a solar‐weighted broadband reflectivity of 93.3%. At the same time, intrinsic molecular vibrations of C─O and C─O─C bonds in the cellulose backbone give rise to a high average emissivity of 92.6% within the atmospheric window (Figure S15). Benchmarking against existing mulching materials (Figure S16) highlights the superior performance of SRCM. Its solar‐weighted reflectivity is nearly twice that of commercial reflective mulches (CRM, 51%) and biodegradable mulches (CBM, 49%), and it far outperforms conventional black mulches (BM, 5%) and translucent white mulches (WM, 14%). Although a common cellulose backbone accounts for similar infrared spectra, the reduced solar reflectivity of CBM arises from solar‐absorbing components such as lignin [59]. In addition, the diffuse scattering behavior of SRCM sustains high solar reflectivity over a wide range of incident angles (Figure 3i). This angular robustness is attributed to its hierarchical architecture and results in minimal solar heating. Reflectivity also increases with mulch thickness, reaching 90% at 350 µm and saturating above 92.5% for thicknesses greater than 500 µm (Figure S17). Achieving such high reflectivity at moderate thickness improves both material efficiency and cost‐effectiveness. Optical spectra of SRCMs with different CNF contents were further evaluated (Figure S18), and SRCM‐5% was identified as offering the optimal balance between mechanical robustness and optical performance.

The practical viability and operational longevity of SRCM in agricultural settings require strong resistance to environmental stressors, which we subsequently evaluated. One primary challenge is moisture from precipitation, which can infiltrate porous mulches, increase solar absorption via wetting, and accelerate mechanical degradation. To mitigate these effects, we introduced surface hydrophobicity by modifying SRCM with silica nanoparticles. This treatment yielded a near‐superhydrophobic surface with a water contact angle of 149.2°, while preserving the material's original optical performance (Figures S19 and S21). Furthermore, the hydrophobic durability was evaluated (Figure S20). The CA remained above 125° after 70 cm of abrasive paper abrasion under 2.6 kPa, stayed near its initial value of ∼149° after 24 h of immersion at pH 2, and remained near the near‐superhydrophobic threshold after continuous UV exposure, supporting the long‐term field deployment of SRCM. Moreover, given the amount of silica used is very small and its chemistry is identical to that of natural sand, any residual remaining after degradation is expected to pose negligible risk of soil contamination. SRCM also shows excellent environmental durability over its operational lifetime as a surface mulch. Its reflectivity remains nearly unchanged after 24 h of water immersion (Figure S22), confirming strong anti‐swelling stability under rainy conditions. Moreover, following accelerated UV aging that corresponds to six months of outdoor exposure, its reflectivity spectrum displayed no observable deterioration (Figure S23), demonstrating that the optical performance is robust under prolonged aboveground exposure and ensuring reliable in‐field performance. Moreover, in contrast to conventional plastic‐based mulches that are essentially air‐impermeable, SRCM exhibits favorable air permeability (see Table S4 and Experimental Section), thereby ensuring natural gas exchange within the plant root zone and favoring healthy crop growth.

### 2.4 Cooling and Water Saving Performance of SRCM

**FIGURE 4:**
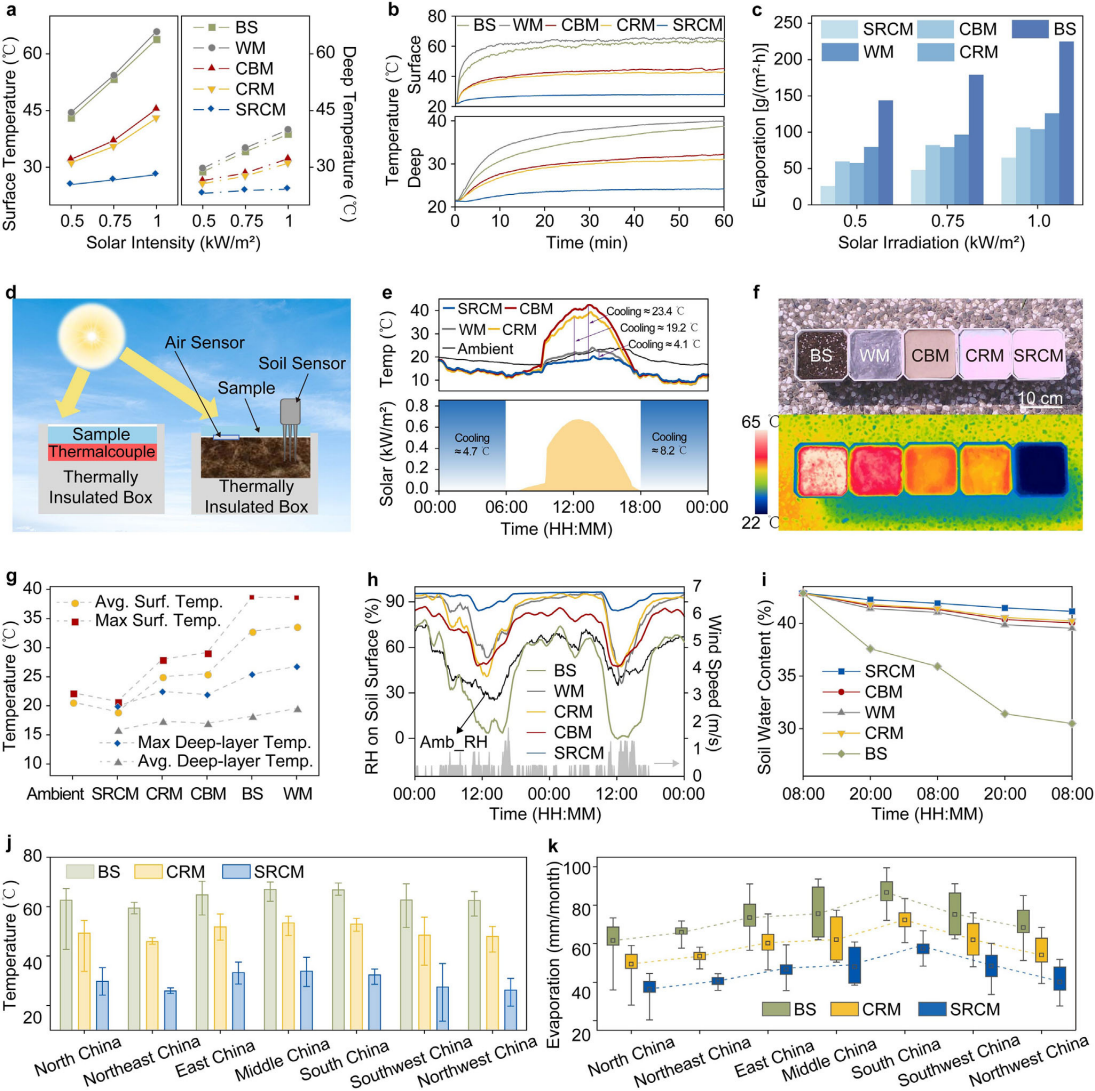
Cooling and water‐saving performance of SRCM. (a) Surface (2 mm) and subsurface (3 cm) Temperatures under different coverings at varying solar irradiance intensities. (b) Dynamic temperature variation of the soil surface and subsurface covered with different mulches under 1 kW light intensity over one hour. (c) Evaporation fluxes across irradiance levels for the various covering treatments. (d) Schematic of the outdoor experimental setup for sub‐ambient temperature management (left) and cooling/water‐retention performance (right). (e) Surface temperature of different mulches over 24 h in Hong Kong (January 3, 2025), with ambient temperature as a reference. (f) Thermal imaging comparison of soil surfaces covered with different mulches in an outdoor environment. (g) 48‐h Summary of maximum and mean surface and subsurface temperatures in Hong Kong (January 17–19, 2025). (h) Measurements of soil surface humidity covered with different mulches over 48 h in Hong Kong (January 17–19, 2025), alongside corresponding ambient relative humidity and wind speed. (i) Measurements of soil water content under different mulches over 48 h in Hong Kong (January 24–26, 2025). (j) Regional summer peak soil temperatures for BS, CRM, and SRCM across China's Seven Divisions. (k) Regional monthly evaporation for BS, CRM, and SRCM based on 2023 Gleam Satellite Data.

The core agricultural functions of SRCM, specifically its ability to mitigate soil heat stress and conserve soil moisture, were evaluated under both indoor and outdoor conditions, beginning with controlled indoor experiments to quantify its cooling and moisture‐retention performance (Figures S24 and S25). SRCM effectively suppressed heat accumulation across a wide range of irradiance levels (Figure 4a). Under peak solar irradiance (1 sun), it maintained a surface temperature of 28°C and a 3 cm subsurface temperature of 24°C. These values correspond to substantial reductions of 36°C at the surface and 14°C at the subsurface relative to bare soil. In comparison, CRM‐ and CBM‐covered surfaces reached 45°C, with subsurface temperatures of 32°C, which are 8°C–17°C higher than those under SRCM. Thermal imaging further confirmed that SRCM exhibited the lowest apparent temperature among all tested mulches (Figure S26). Notably, the use of WM resulted in soil temperatures that were even higher than those measured under bare soil. This heating is consistent with a greenhouse mechanism, where high visible and near‐infrared transmittance deposits solar energy directly into the soil while mid‐infrared opacity traps the outgoing longwave radiation. In contrast, the high solar reflectivity of SRCM greatly reduces solar heat input, and this effect is sufficiently strong to sustain cooling even when the atmospheric thermal emission channel is restricted. SRCM also showed rapid and persistent cooling. Under continuous irradiation, it reached a steady‐state temperature below 25°C within 10 min and maintained this value throughout a 60‐min test. (Figure 4b and Figure S27). Beyond cooling, SRCM was highly effective in conserving water by suppressing evaporation at all irradiance levels (Figure 4c). At 1.0 kW/m^2^, water mass loss was limited to 65 g m^−^^2^ h^−^^1^, corresponding to a reduction of more than 71% compared with bare soil and outperforming other commercial mulches by up to 45%.

SRCM was then evaluated outdoors to assess its performance under realistic field conditions (Figure 4d and Figure S28). Under peak solar irradiance of 0.65 kW/m^2^, it achieved pronounced sub‐ambient cooling at noon. SRCM surface temperature was 4.1°C below ambient and 23.4°C and 19.2°C lower than those of CBM and CRM, respectively (Figure 4e). Infrared thermography (Figure 4f) corroborated this effect, showing SRCM surface near 22°C, whereas bare soil and WM surfaces exceeded 60°C. To further address the influence of variable field conditions, we performed a theoretical net cooling power analysis as a function of the surface‐to‐ambient temperature difference and the overall heat transfer coefficient (Figure S29). The results show that, although the attainable cooling power decreases with increasing parasitic heat exchange, SRCM retains a positive net cooling capability over a practically relevant range of environmental perturbations, consistent with the outdoor measurements. A subsequent 48‐h soil test further confirmed this cooling efficacy (Figure 4g and Figures S30 and S31). The maximum soil surface temperature under SRCM was only 20.8°C, which was 7.3°C–18.0°C lower than the corresponding temperatures under CRM, CBM, WM, and bare soil. Subsurface temperatures displayed the same trend. This substantial temperature reduction directly lowers the saturation vapor pressure at the soil–SRCM interface, thereby diminishing the thermodynamic driving force for evaporation and enhancing water retention (Figure 4h,i). As a result, even at midday, SRCM maintained near‐surface relative humidity above 80%, compared with 50%–65% for commercial mulches and less than 20% for bare soil. Over 48 h, bare soil exhibited a 28.9% reduction in water content, a critical depletion sufficient to drop below the permanent wilting point and induce severe plant water stress [60]. By contrast, SRCM‐covered soil maintained volumetric water content above 40%, with only a 4.1% decline, corresponding to an 85.8% suppression of evaporative water loss relative to bare soil.

The thermal and moisture‐regulation performance of SRCM was further assessed through simulations covering China's seven major geographical regions [61] (Figure S32), in order to evaluate regional effectiveness and guide deployment strategies. Using bare soil and CRM (the best‐performing commercial mulch in our preliminary tests) as benchmarks, the simulations show that SRCM consistently limits summer peak soil temperatures to around 33°C. This corresponds to an average reduction of 34°C relative to bare soil and 20°C relative to CRM (Figure 4j and Figure S33), thereby bringing soil temperatures much closer to the 15°C–30°C optimal range for most crops. This thermal regulation translates directly into substantial water savings. Analysis based on 2023 GLEAM satellite data indicates large reductions in monthly evaporation under SRCM across all climatic zones [62] (Figure 4k, Figure S34 and Table S5). In humid South China, SRCM reduced evaporation by 33% compared with bare soil and by 20% compared with CRM. In the arid Northwest, the reductions were even greater, reaching 42% and 27%, respectively. These regional differences arise from distinct underlying mechanisms. In humid regions, the dominant benefit is surface cooling, which lowers the vapor pressure deficit between soil and air and thus suppresses latent heat flux. In arid regions, the high solar reflectivity and thermal emissivity of SRCM play a central role by limiting the net energy available for phase change. These insights enable climate‐specific deployment strategies. In arid regions such as Northwest China, continuous application of SRCM throughout the hot season is optimal for maximizing both cooling and water conservation. In humid East and South China, targeted short‐term use during extreme heat events can effectively moderate peak soil temperatures and stabilize soil moisture. In the central transitional zone, a flexible deployment strategy is recommended, where SRCM is applied during heat‐sensitive growth stages or in response to defined environmental thresholds and is coordinated with irrigation scheduling.

### 2.5 Outdoor Plantation Experiment

**FIGURE 5:**
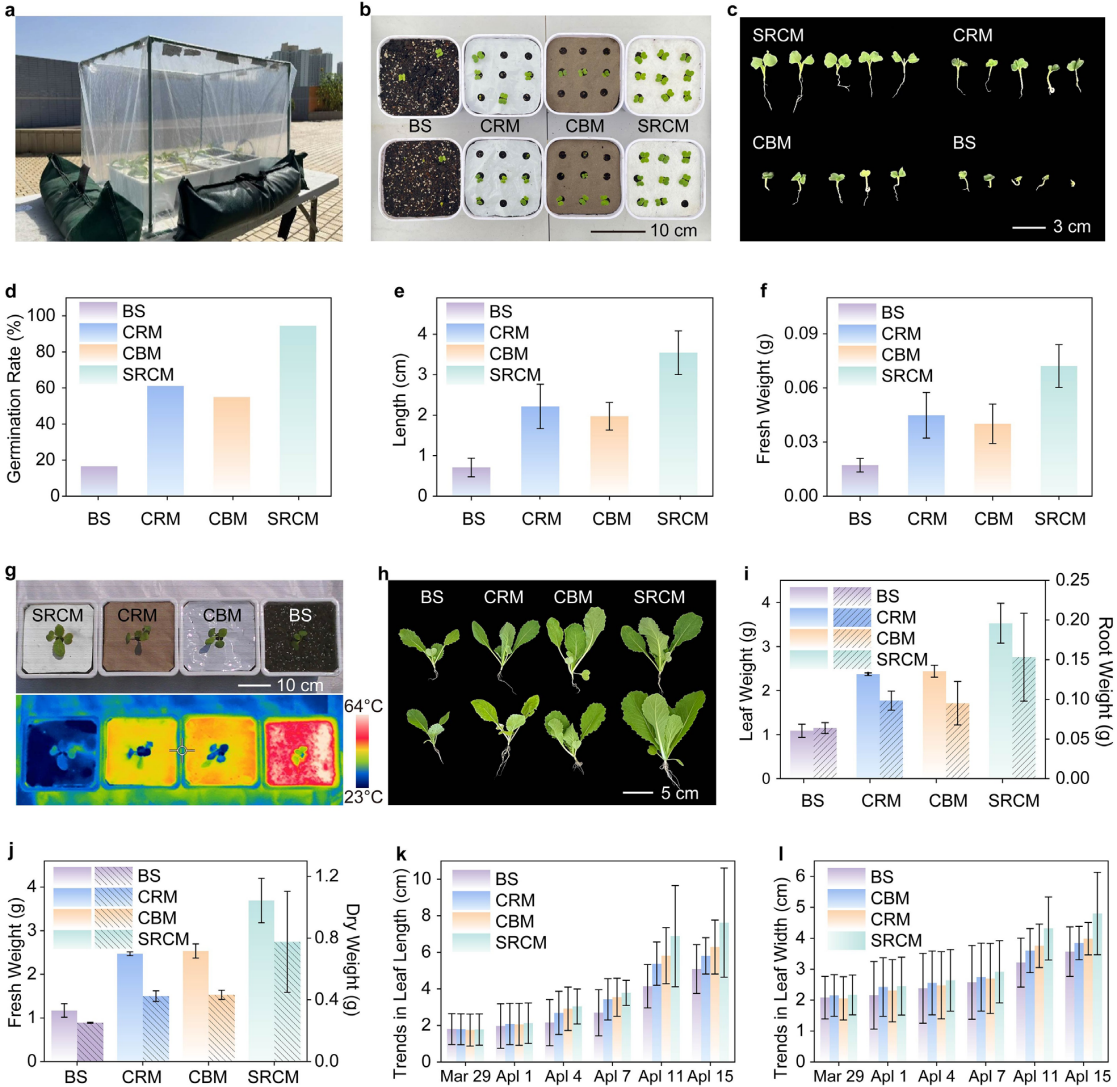
Effect of different mulches on early‐stage crop growth. (a) The experimental setup, showing an open‐top greenhouse chamber (1.1 m × 0.7 m × 0.6 m) containing eight pots (14.2 cm side length), weighted to mitigate wind effects. (b) Photograph of germination status at 10 days after sowing (DAS) with a seed depth of 1 cm. (c) Side view of seedlings under different mulches at 10 DAS. (d) Germination rate under different mulches. (e) Seedling length under different mulches (f). Seedling fresh weight under different mulches (g). Visible and infrared photographs of post‐emergence seedlings taken at midday. (h) Side view of plants at 20 days post‐emergence under different mulches. (i) Leaf and root weight of plants under different mulches. (j) Fresh and dry weight of plants under different mulches. (k, l) Time course of post‐emergence leaf length and width for plants under different mulches.

The agronomic performance of SRCM was evaluated in an outdoor planting experiment using bok choy as a model crop. An open‐top chamber system was used to compare four treatments under identical ambient conditions, with SRCM, CRM, CBM, and bare soil serving as the test groups. (Figure 5a and Figure S35). The cooler and more humid microclimate created by SRCM was associated with faster germination and improved early seedling establishment. Ten days after sowing, seedlings grown under SRCM displayed clearly higher vigor and more uniform emergence than those in the other treatments. Quantitatively, the germination rate under SRCM reached 95%, which exceeds the typical agricultural benchmark of 70%–90% [60]. This germination rate is 5.3 times higher than the 15% observed for bare soil and represents relative improvements of 61% and 54% compared with CRM and CBM, respectively (Figure 5b–d). The mean sprout length under SRCM was 3.5 cm, whereas bare soil, CRM, and CBM produced average lengths of 0.7, 2.2, and 2.0 cm, respectively. Fresh biomass followed a similar trend, with SRCM‐grown seedlings reaching 0.077 g, which is more than four times the value for bare soil (0.018 g) and about 80% higher than those for both CRM and CBM (Figure 5f).

To assess post‐emergence growth, eight size‐matched 10‐day‐old seedlings were transplanted into a controlled greenhouse, where a 2 cm central opening in each cover allowed shoot extension. Midday thermography showed that the surface temperature under SRCM was 23°C, much lower than that of bare soil at 64°C and of CBM and CRM at about 61°C. At the same time, the leaf temperature of seedlings grown with SRCM remained at a cool 26°C (Figure 5g). This foliar cooling is attributed to vigorous transpiration, supported by the high soil moisture maintained beneath SRCM, together with the passively cooled mulch surface [63]. After 20 days, these favorable thermo–hydrological conditions led to substantial gains in plant biomass. Leaf mass of SRCM‐mulched plants reached 3.52 g, corresponding to a 226% increase over the bare soil control and a 48%–49% increase over both CRM and CBM (Figure 5h). Root biomass showed a similar trend. Plants grown with SRCM produced 2.4 times more root mass than those on bare soil and about 60% more than those under the other mulches (Figure 5i). Overall, the fresh mass of SRCM plants was 3.69 g, more than three times that of bare soil plants at 1.17 g, while dry mass reached 0.77 g, compared with 0.25 g for the control. Relative to CBM and CRM, SRCM increased fresh mass by 46%–49% and dry mass by 79%–83% (Figure 5j). Collectively, these results show that SRCM markedly enhances plant growth and biomass accumulation throughout the post‐emergence stage.

Growth dynamics were further monitored through periodic measurements of leaf length and width in each treatment group (Figure 5k–l and Figure S36). During the first five days, leaf dimensions changed little across all treatments, which is consistent with the initially uniform soil conditions. Thereafter, growth trajectories diverged. Seedlings in the SRCM group displayed the fastest growth and reached the largest final leaf size, with lengths 1.68 times greater and widths 1.59 times greater than those of the bare soil control. Leaves in the SRCM group were also 43%–47% longer and 36%–44% wider than those in the CRM and CBM groups. Notably, the two conventional mulches showed distinct growth responses relative to each other. CRM slightly improved germination, whereas CBM was more favorable for post‐emergence growth, although both consistently outperformed bare soil. This nuanced difference indicates that, under comparable photothermal conditions, nonthermal factors such as soil water retention and gas permeability become important secondary determinants of crop growth [64]. Overall, these results show that the superior hydrothermal regulation provided by SRCM establishes a more favorable soil microenvironment, which in turn accelerates germination, enhances early vegetative growth, and substantially increases biomass.

### 2.6 Recyclability, Biodegradability, Biosafety, and Carbon Reduction of SRCM

**FIGURE 6:**
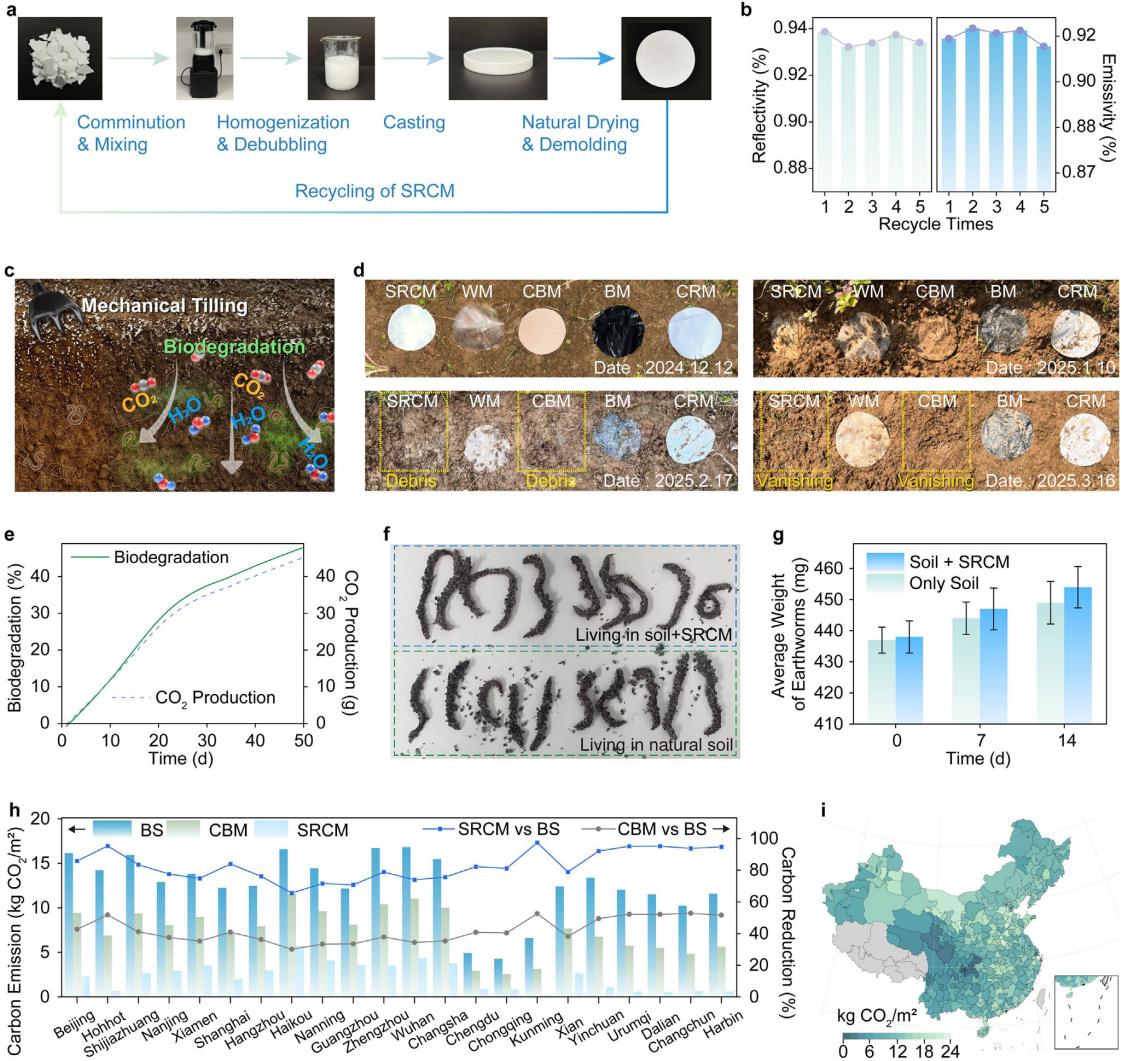
Recyclability, biodegradability and biosafety of SRCM. (a) Schematic illustration of the recycling process for SRCM. (b) Effect of multiple recycling cycles on the optical properties of SRCM. (c) Schematic illustration of the biodegradation mechanism of SRCM in a soil environment. (d) Degradation of SRCM in a real soil environment. (e) Degradation of SRCM under composting conditions. (f) Ecotoxicity test comparing the survival of earthworms in soil amended with SRCM degradation products (top) versus untreated soil (bottom). (g) Change in the average body mass of earthworms during the ecotoxicity test. (h) City‐level greenhouse carbon emissions under BS, CBM and SRCM and corresponding reductions relative to BS. (i) Spatial distribution of modeled greenhouse carbon‐emission reductions achieved by SRCM relative to bare soil across China.

The practical utility and service longevity of SRCM are substantially enhanced by its inherent recyclability, a feature that enables the straightforward reprocessing of material following accidental fragmentation or at the end of life. This reprocessing capability is a direct consequence of the extensive hydrogen bonding network within the material's structure, enabling the reprocessing of the material through a facile aqueous method that involves depolymerizing the material into a slurry and subsequently reconstituting it via casting and ambient drying (Figure 6a). The robustness of this recycling route was verified by subjecting SRCM to five consecutive recovery and regeneration cycles and monitoring its key optical properties. Across all cycles, both solar reflectivity and thermal emittance remained essentially unchanged, with no measurable degradation (Figure 6b).

SRCM offers a sustainable end‑of‑life pathway through complete biodegradation, avoiding the long‑term environmental burden associated with persistent plastic waste. After use, it does not require manual retrieval. Instead, SRCM can be directly incorporated into the soil through conventional agricultural operations such as tilling, where it is gradually transformed into organic soil matter (Figure 6c). In situ soil burial assays under simulated field conditions confirmed the superior biodegradability of SRCM. Its degradation clearly exceeded that of conventional plastic mulches and was comparable to that of CBM. SRCM underwent complete disintegration and assimilation within about three months (Figure 6d), a timeframe that matches typical fallow periods and therefore does not interfere with the subsequent planting season. In contrast, the plastic mulches showed no visible change over the entire observation period, indicating negligible biodegradation and a significant risk of long‐term environmental accumulation. The rapid degradability of SRCM was further quantified using a standardized composting test, in which CO_2_ evolution was monitored as a proxy for mineralization (experimental setup detailed in Figure S37) [65]. Under these conditions, SRCM reached a degradation level of almost 49% within 52 days (Figure 6e). This performance surpasses that of a recently reported ethyl cellulose‐based PRC mulch, which exhibits less than 5% degradation over one month [17]. This high biodegradation rate arises from the intrinsic chemistry and supramolecular organization of neat cellulose. The abundance of accessible hydroxyl groups and the hydrophilic surface promote water absorption and microbial colonization, allowing cellulolytic microorganisms to secrete enzymes that attack chain ends [66]. In addition, neat cellulose does not contain hydrophobic substituents, unlike ethyl cellulose, which reduces kinetic barriers to depolymerization and subsequent mineralization [51]. Notably, these biodegradation pathways operate most efficiently under burial or composting conditions, where continuous soil contact, elevated moisture, and high microbial activity are sustained. By contrast, during normal use as a surface mulch, these drivers are greatly attenuated [67], and SRCM maintains stable optical and mechanical performance over the crop growth period.

While SRCM's complete biodegradation is environmentally desirable, its constituent chemical species are released into the surrounding environment during this process. In principle, such degradation products could transiently disturb soil homeostasis and influence crop performance [68, 69], so an ecotoxicological assessment is necessary to confirm environmental safety under agricultural conditions. To this end, we evaluated the ecotoxicological effects of SRCM degradation on soil health using earthworms as bioindicators. Over a 14‐day exposure period, no mortality was observed in SRCM‐treated group. In addition, earthworms in this group showed a slight increase in average body weight compared with the untreated control (Figures 6f–g and Figure S38) [70]. These results indicate that SRCM degradation is not harmful to soil fauna and may even provide modest benefits to the soil ecosystem, supporting its suitability for safe agricultural application.

Beyond its end‐of‐life advantages, SRCM also promotes agricultural sustainability during use by enabling substantial carbon reductions. This benefit arises from its PRC capability, which can be exploited in controlled environments such as greenhouses to lower cooling energy demand and thereby reduce carbon emissions. To quantify this potential, we modeled a standard Venlo‐greenhouse in EnergyPlus and compared three soil surface conditions, SRCM, CRM, and bare soil. The indoor air temperature was constrained between 15°C and 30°C to support optimal crop growth, and cooling was provided by an idealized HVAC system with a coefficient of performance of 3.0 (**model configuration detailed** in Table S6 and Figure S39). The simulations show that covering greenhouse soil with SRCM markedly reduces carbon emissions across all evaluated cities (Figure 6h). On average, SRCM achieved a carbon abatement of 10.3 kg CO_2_/m^2^, corresponding to an 83% reduction relative to bare soil. Compared with CRM, SRCM provided an additional abatement of 5.15 ± 1.38 kg CO_2_/m^2^, which represents a further 41.2% mitigation. Spatially, the model reveals that mitigation is most concentrated in major agricultural belts and regions with high cooling demand like eastern and southern regions, with site‐specific abatement reaching up to 24 kg CO_2_/m^2^ relative to bare soil (Figure 6i). These carbon reductions are directly linked to corresponding energy savings (Figures S40 and S41). Together, these results indicate that prioritizing SRCM deployment in energy‐intensive agricultural regions can effectively curb operational carbon emissions and enhance the energy carbon efficiency of controlled environment agriculture, thereby supporting low‐carbon and sustainable agricultural development.

## 3 Conclusion

This work introduces a closed‐loop, all‐biomass PRC mulch derived from agricultural residues for root‐zone thermal and moisture management. Application‐grade mechanical robustness is achieved by integrating nanofibers extracted from the same feedstock, avoiding the use of chemical crosslinkers. Enabled by an energy‐efficient, hydrogen‐bond‐driven self‐assembly route in water, the mulch forms a hierarchically porous, multiscale cellulosic network that enhances broadband solar scattering while leveraging the intrinsic mid‐infrared emissivity of biomass for effective daytime cooling. In field deployment, SRCM reduces heat loading to the soil and suppresses evaporation, thereby improving soil temperature and moisture conditions and promoting early‐stage crop establishment. Its degradable and recyclable design supports residue‐material‐soil cycling within agricultural practice and shows no detectable adverse impact on soil microbial activity. Beyond open‐field use, SRCM also demonstrates reduced greenhouse‐gas emissions relative to commercial reflective mulches in protected cultivation, underscoring its low‐carbon potential. Overall, this study establishes a scalable aqueous manufacturing and design paradigm that integrates resource circularity, environmental compatibility, and PRC, offering a sustainable pathway to maintain productivity under intensifying heat and drought stress.

Looking forward, sustainable radiative cooling mulches (SRCMs) are poised to evolve from passive cooling materials into multifunctional, climate‐adaptive agricultural interfaces. A key challenge lies in utilizing solar‐reflective cooling to precisely regulate the radiative and thermal microenvironment during early‐stage crop establishment, thereby alleviating heat stress and supporting subsequent growth. Equally critical is the development of stimuli‐responsive biomass architectures capable of dynamically regulating emissivity or reflectance in response to temperature, humidity, or irradiance, thereby mitigating overcooling under low‐temperature or nighttime conditions. Integrating radiative cooling with atmospheric water harvesting or controlled moisture release further offers a promising pathway to enhance drought resilience while maintaining soil health, material sustainability, and residue circularity. Field translation will additionally require durable optical and mechanical performance under UV exposure, soil‐contact aging, and dust accumulation, underscoring the need for standardized accelerated‐aging and multi‐season evaluation protocols. More importantly, bridging material‐level performance with agronomic outcomes will demand system‐level validation through life‐cycle assessment, techno–economic analysis, and multi‐climate field trials. Addressing these interconnected challenges will be essential for advancing SRCMs from proof‐of‐concept materials to deployable, climate‐resilient agricultural technologies.

## 4 Experimental Section

The detailed experimental procedures are provided in the Supporting Information.

## Author Contributions

**Hao Li** and **Dong Lv** contributed equally to this work. Conceptualization: Hao Li, Dong Lv, **Chi Yan Tso**; Methodology: Hao Li, Dong Lv, **Yang Fu**, **Cancheng Jiang**, **Wenqi Wang**, **Ze Li**; Investigation: Hao Li, Dong Lv, Cancheng Jiang, Wenqi Wang, Ze Li, **Lin Liang**, **Jiayu Du**, **Jie Tan**, **Yihao Zhu**, **Wenjie Liu**, **Lamfeddal Kouisni**, **Kaixin Lin**, Chi Yan Tso; Funding acquisition: Chi Yan Tso; Supervision: Chi Yan Tso; Writing – original draft: Hao Li, Chi Yan Tso; Writing – review & editing: Dong Lv, Yang Fu, K.L., Chi Yan Tso

## Conflicts of Interest

The authors declare no conflicts of interest.

## Supporting information




**Supporting File**: advs75987‐sup‐0001‐SuppMat.docx.

## Data Availability

The data that support the findings of this study are available from the corresponding authors upon request.
