# Supplementary material for: Closed‐Loop Radiative Cooling Mulch Upcycled From Agricultural Residues for Efficient Soil Heat–Water Stress Mitigation
Source: Adv Sci (Weinh). 2026 Jun 9;13(49):e75987. doi: 10.1002/advs.75987 (PMC13336867; doi:10.1002/advs.75987)
Supplement: Supplementary file 1 [file ADVS-13-e75987-s001.docx]

Supporting Information

**Closed-Loop Radiative Cooling Mulch Upcycled from Agricultural Residues for Efficient Soil Heat–Water Stress Mitigation**

*Hao Li^1†^, Dong Lv^1†^, Yang Fu^1^, Cancheng Jiang^1^, Wenqi Wang^1^, Ze Li^1^, Lin Liang^1^, Jiayu Du^1^, Jie Tan^1^, Yihao Zhu^1^, Wenjie Liu^2,3^, Lamfeddal Kouisni^4^, Kaixin Lin^1^*, Chi Yan Tso^1^**

**Affiliations:**

^1^School of Energy and Environment, City University of Hong Kong, Hong Kong SAR, 999077, China.

^2^School of Energy and Environmental Engineering, University of Science and Technology Beijing, Beijing, 100083, China

^3^Shunde Innovation School, University of Science and Technology Beijing, Foshan 528399, China

^4^African Sustainable Agriculture Research Institute (ASARI), College of Agriculture and Environmental Science, University Mohammed VI Polytechnic (UM6P), Laâyoune, Morocco.

^†^These authors contributed equally to this work.

*Corresponding author. Email: [chiytso@cityu.edu.hk](mailto:chiytso@cityu.edu.hk) (C.Y.T.); [Johnny.lin@cityu.edu.hk](mailto:Johnny.lin@cityu.edu.hk) (K.L.)

**Experimental Section**

**Materials.** Waste dried corn leaves were collected from local farmland. Sodium hydroxide (NaOH, ≥98%), sodium sulfite (Na₂SO₃, ≥98%), hydrogen peroxide (H₂O₂, 30 wt.% aqueous solution), 2,2,6,6-tetramethylpiperidine-1-oxyl radical (TEMPO, 98%), sodium bromide (NaBr, ≥99%), hydrochloric acid (HCl, 37 wt.%), and anhydrous ethanol (≥99.5%) were purchased from Sigma-Aldrich. Sodium hypochlorite solution (NaClO, analytical grade, available chlorine content ≥8%) was purchased from Aladdin Biochemical Technology Co., Ltd. (Shanghai, China). Deionized (DI) water was used in all experiments.

**Extraction of Micron-scale Cellulose from Waste Maize Leaves.** The cellulose extraction process was designed for the selective removal of lignin and hemicellulose ^[1]^. First, dried corn leaves were chopped and immersed in an alkaline delignification solution containing sodium hydroxide and sodium sulfite. The mixture was then boiled for 3 hours under continuous stirring. Upon completion of the reaction, the cellulose residue was collected via vacuum filtration and washed repeatedly with hot deionized water until the filtrate reached neutrality, ensuring the removal of most chemical residues. Subsequently, the obtained crude cellulose was subjected to a bleaching treatment by boiling in a hydrogen peroxide solution for an additional 3 hours. After the yellow color of the fibres had completely disappeared, the sample was retrieved, washed with cold deionized water until neutral, and finally dispersed in ethanol for storage and subsequent use.

**Preparation of Nanocellulose via TEMPO-mediated Oxidation.** Nanocellulose was prepared using a TEMPO-mediated oxidation method ^[2]^. Initially, the micron-scale cellulose extracted in the previous step was prepared as a 1.0 wt.% aqueous suspension. Catalytic amounts of TEMPO (0.016 g per gram of cellulose) and sodium bromide (0.1 g per gram of cellulose) were sequentially added to the suspension. The mixture was stirred thoroughly in an ice-water bath, after which a sodium hypochlorite solution (5 mmol per gram of cellulose) was added dropwise to initiate the oxidation reaction. Throughout the process, the pH of the system was maintained at 10.0 by the continuous addition of a sodium hydroxide solution. The completion of the carboxylation reaction was indicated by a stable pH that no longer decreased. Subsequently, the reaction was quenched with a small amount of ethanol, and the pH of the suspension was adjusted to neutral using dilute hydrochloric acid. Byproducts and unreacted reagents were thoroughly removed through repeated cycles of centrifugation and washing with deionized water. Finally, the purified product was processed via high-pressure homogenization to yield a uniform and transparent 1.0 wt.% suspension of cellulose nanofibers (CNFs).

**Fabrication of the Sustainable Radiative Cooling Mulch (SRCM).** First, the cellulose microfibers (CMF) extracted in Section 2.2 was dispersed in deionized water to prepare a 1.0 wt.% suspension. This was achieved by processing the mixture for 5 minutes at 15,000 rpm using a high-speed blender (Fronton, USA). Subsequently, this CMF suspension was blended with the CNF suspension from Section 2.3 at various dry weight ratios (97.5:2.5, 95:5, 92.5:7.5, and 90:10). A control sample containing only pure cellulose microfibers (100:0) was also prepared. The thoroughly mixed slurries were then cast into molds and allowed to dry naturally via evaporation for 5 days under ambient conditions (approximately 25 °C) in a well-ventilated environment, resulting in the formation of self-supporting films. Based on the CNF content, the resulting mulch samples were designated SRCM-0%, SRCM-2.5%, SRCM-5%, SRCM-7.5%, and SRCM-10%, respectively.

**Materials Characterization.** Optical properties, including reflectance and transmittance within the solar spectral range (0.25 to 2.5 µm), were characterized using a UV-Vis-NIR spectrophotometer (LAMBDA 1050+, PerkinElmer). The instrument was equipped with a 150 mm integrating sphere, and Spectralon was used as the diffuse reflectance standard. Optical properties in the infrared range (2.5 to 25 µm) were obtained using a Fourier-transform infrared (FTIR) spectrometer (IRAffinity-1S, Shimadzu) fitted with a gold-sputtered integrating sphere (MID-IR IntegratIR, PIKE), using a diffuse gold surface as the reference. The average reflectance was calculated using the following formula:

$$\begin{aligned} \bar{R}=\frac{\int_{\lambda_{1}}^{\lambda_{2}} I_{AM}\left( \lambda\right)\cdot R\left( \lambda\right)d\lambda}{\int_{\lambda_{1}}^{\lambda_{2}} I_{AM}\left( \lambda\right)d\lambda}\#\left( 1 \right) \end{aligned}$$

where $I_{AM}\left( \lambda\right)$ is the standard AM1.5 solar spectrum and $R\left( \lambda\right)$ is the measured spectral reflectance. The average solar reflectance and average visible reflectance are calculated for the wavelength range $\lambda_{1}$ - $\lambda_{2}$ of 0.25 to 2.5 μm. Similarly, the average transmittance is calculated using the following formula:

$$\begin{aligned} \bar{\tau}=\frac{\int_{\lambda_{1}}^{\lambda_{2}} I_{AM}\left( \lambda\right)\cdot\tau\left( \lambda\right)d\lambda}{\int_{\lambda_{1}}^{\lambda_{2}} I_{AM}\left( \lambda\right)d\lambda}\#\left( 2 \right) \end{aligned}$$

where $\tau\left( \lambda\right)$ is the measured spectral transmittance. The absorptance (α) was calculated as α = 1 - $\bar{R}$ - $\bar{\tau}$. For the SRCM, infrared transmittance was negligible (≈ 0), thus simplifying the infrared absorptance calculation to 1 - $R$.Surface morphology was characterized by field-emission scanning electron microscopy (FESEM, Zeiss SIGMA 500) at an accelerating voltage of 8–10 kV. Fourier-transform infrared (FTIR) spectra were acquired with a PerkinElmer Spectrum 100 instrument. X-ray diffraction (XRD) analysis was performed using a Bruker diffractometer over a 2θ range of 5–80° with a 0.02° step size. Water contact angles were determined by depositing a 10 μL deionized water droplet onto the material surface and capturing its image with a high-speed camera (Phantom, Micro C110) once stable. Tensile properties were measured on a universal testing machine (Instron 5967, USA) per ASTM D882. For accelerated aging, samples were exposed for two weeks in a UV weathering chamber (QUV, Q-Lab) with UVA-340 lamps at 60 °C ^[3]^. Their optical properties were then re-measured to assess performance degradation. During cooling tests, surface temperatures were monitored using a high-resolution thermal camera (FLIR E6). The camera's emissivity was calibrated to the material's value (ε ≈ 0.92) to ensure measurement accuracy.

**Biodegradation Test.** The biodegradability of the material was evaluated under controlled composting conditions by measuring the evolved carbon dioxide, in accordance with the GB/T 19277.1-2011 standard (equivalent to ISO 14855-1:2005). The compost was derived from the organic fraction of municipal solid waste, which had been previously composted for three months. The experiment was conducted in 2.5 L quartz glass vessels. The dry weight ratio of compost to the test or reference material was approximately 6:1. The system was continuously aerated with CO₂-free air, ensuring the oxygen concentration in the exhaust gas remained above 6%. The test was performed at 58 ± 2 °C under dark conditions, and the amount of evolved CO₂ was determined by titration. Thin-layer chromatography (TLC) grade cellulose served as the positive reference material. The average biodegradation rate was calculated based on the cumulative amount of CO₂ evolved throughout the test and the loss of volatile solids at the experiment's conclusion. Throughout the test period, CO₂ evolution was periodically recorded for the test material, the reference sample, and a blank control (compost only). Furthermore, the designed SRCM was evaluated via a soil burial test. The test was conducted in Hong Kong (22.28°N, 114.17°E), commencing on December 12, 2024. Circular samples (5 cm in diameter) of the SRCM, CRM, CBM, BM, and translucent WM were buried in natural soil. The overlying soil was removed on days 29, 67, and 95 to observe the morphological changes of the samples. By the final observation, the SRC and CB samples had almost completely degraded, leaving no visible residue.

**Biosafety Test.** The biosafety of the mulch degradation products was evaluated using the earthworm Eisenia foetida (average body weight: 300–500 mg) as the biological model. To prepare the test medium, 2.4 g of the SRCM degradation product was mixed with 37.6 g of quartz sand and 2360 g of artificial soil. This dry mixture was then hydrated with 840 g of pure water, resulting in a final concentration of 1000 mg of degradation product per kg of the dry soil mixture. The test was conducted over a 14-day period with a blank control group (containing no SRCM degradation products) and an experimental group at a concentration of 1000 mg/kg. For each replicate, 750 g of the prepared test medium was placed into a 1 L beaker, and 10 earthworms were introduced. The beakers were sealed with a breathable membrane and maintained in an incubator for the duration of the experiment. On day 7 and day 14, the earthworms were observed, and their body weight and mortality were recorded.

**Hydrophobic Treatment.** Isopropanol was thoroughly mixed with nano-silica particles and sealed for stirring for 1 hour. The resulting mixture was then evenly applied to the surface of the potted SRCM. Subsequently, the mulch was dried at 50 °C, during which isopropanol rapidly evaporated, leaving a uniform nano-silica layer. Nano-silica, being colorless and transparent, had minimal impact on the optical properties of SRCM. Moreover, silica is a primary component of sand in soil, and its usage was negligible. Thus, even if degraded into the soil after application, it would not cause environmental pollution. The contact angle was measured by gently placing a 10 μL droplet of deionized water on a horizontal surface and capturing images with a high-speed camera (Phantom, Micro C110).

**Durability Testing and Evaluation of the Hydrophobic Treatment**. To comprehensively evaluate the robustness of the hydrophobic treatment for SRCM under realistic service conditions, three complementary durability tests were designed to emulate representative degradation scenarios encountered during practical outdoor use, including mechanical wear, chemical corrosion, and prolonged ultraviolet exposure. To simulate the mechanical damage that the coating may suffer during handling, contact, and service, an abrasion test was performed. The sample was placed face-down onto 1000-grit abrasive paper and horizontally dragged along the abrasive paper under a 100 g weight (corresponding to a contact pressure of 2.6 kPa), with an abrasion distance of 10 cm per cycle. The water contact angle (CA) was recorded after successive abrasion cycles to track the evolution of surface hydrophobicity under continuous mechanical wear. To assess the chemical stability of the coating under acid-rain-like corrosive environments that are commonly encountered in outdoor applications, the sample was immersed in an HCl solution (pH = 2) for extended periods. The CA was measured at predetermined time intervals to monitor any deterioration of the hydrophobic performance induced by prolonged acidic exposure. To evaluate the photo-stability of the coating against the continuous ultraviolet irradiation inevitable in outdoor service, an accelerated UV aging test was carried out in a UV weathering chamber maintained at 25 °C (peak emission at 340 nm, UV irradiance of 0.89 W/m²) for 2 weeks. The applied UV dosage corresponds to approximately 4 months of Florida sunshine exposure (annual UV dosage of ~275 MJ·m⁻²), which serves as an internationally recognized benchmark for material durability evaluation. The CA was recorded every 3 days to capture the long-term evolution of the hydrophobic response under sustained UV aging.

**Air Permeability Measurement.** The air permeability of different mulches (WM, BM, CRM, CBM, and SRCM) was measured using a differential-pressure gas permeability analyzer (VAC-V2, Labthink Instruments Co., Ltd., China). Before testing, all samples were conditioned under a controlled environment of 23 ± 2 °C and 50 ± 2% RH. During the measurement, each sample was fixed between the high-pressure and low-pressure chambers of the instrument, and a pressure difference was applied across the film. The gas permeation through the sample was then continuously monitored until a steady state was reached. The air permeability was subsequently calculated and expressed in cm³/(m²·d·Pa). Each sample was tested three times, and the average values are presented in **Supplementary Table 4**.

**Indoor Photothermal Test.** A solar simulator (SS-X, AM1.5G) was used to replicate solar irradiation. Data were collected at 10-second sampling intervals using K-type thermocouples connected to a LabVIEW software and data acquisition system (NI 9213, NI 9201, cDAQ-9174, National Instruments). The placement of the thermocouples within the experimental setup is illustrated in the schematic in Fig. 4a. The evaporation rate was determined by measuring the mass change of the experimental apparatus with an electronic balance. The initial (mᵢ) and final (mₑ) mass of the setup were recorded before and after each solar simulation period.

**Field investigation.** Outdoor thermal measurements were conducted on a sunny day in Hong Kong. For experiments evaluating sub-ambient cooling, a square, shallow cavity (5 cm in length, 0.5 cm in depth) was created on the top surface of a 30 × 30 × 30 cm expanded polystyrene foam box. The SRC cooling mulch, along with other commercial mulches for comparison, was precisely placed on the surface of this cavity. Thermocouples (K-type, ±0.5 °C) were installed underneath each film to measure the cooling temperature. Data were collected at 30-second sampling intervals using a LabVIEW software and data acquisition system (NI 9213, NI 9201, cDAQ-9174, National Instruments). In the field tests assessing the cooling and moisture-retention performance on soil, an air sensor (Fala IOT, S10A, ±0.3 °C) was used to record the ambient air temperature and humidity, while a LoRa soil temperature sensor (Renke, RS-TR-LORA-2, ±0.5 °C) was used to record the soil temperature. To ensure accurate and consistent environmental data collection, a high-precision commercial weather station (YG-BX, Wuhan Yigu Chenyun Technology Co., Ltd.) was used to measure ambient conditions at 1-minute intervals. The recorded parameters included ambient air temperature (±0.3 °C uncertainty), relative humidity (±3% uncertainty), and wind speed (uncertainty of ±0.3 m/s and ±2%). The weather station was positioned within 2 meters of the measurement setup to guarantee it operated under the same environmental conditions as the samples.

**Plant Growth Experiment.** The plant growth experiment focused on two stages: the seeding stage and the post-emergence growth stage. The experiment was conducted in a rooftop garden at City University of Hong Kong. An open greenhouse measuring 1.1 m × 0.7 m × 0.6 m was constructed, containing eight pots with a side length of 14.2 cm. During the seeding stage, nine seeds were sown in each pot, with 8 mm holes punched in the soil-covering film corresponding to the seed positions. Plants were irrigated with clean water every two days. After 10 days, germination was assessed by measuring germination rate, seedling height, fresh weight, and dry weight. In the post-emergence growth stage, one 10-day-old germinated seedling was transplanted into each pot to ensure uniform initial growth conditions across all groups. Irrigation remained bi-daily, while seedling assessments, including measurements of maximum leaf length and width, were conducted every three days. The experiment lasted approximately three weeks. Final measurements included leaf width, leaf length, fresh weight, and dry weight (dry weight was obtained by drying samples in an oven at 65 °C until a constant weight was reached).

**Molecular Dynamics Simulation.** Classical molecular dynamics (MD) simulations were conducted using the Forcite module within Materials Studio. Two distinct model systems were constructed to investigate the effects of modification: the first system comprised a pristine cellulose chain paired with a carboxyl-modified cellulose chain, while the second system consisted of two unmodified cellulose chains serving as a control. Each cellulose molecule was composed of six glucose units. The COMPASS III force field was employed for all simulations, with atomic charges assigned according to the default force-field charge scheme. A cutoff distance of 12.5 Å was applied to calculate non-bonded interactions.

The simulation protocol consisted of a two-stage equilibration process. Initially, each system underwent an NPT (constant number of particles, pressure, and temperature) simulation at 298 K and 0.0001 GPa for a total duration of 1 ns. This phase was divided into two 0.5 ns segments, utilizing a time step of 2 fs for the first segment and 1 fs for the second. Temperature was maintained using the Nosé thermostat, while pressure was regulated via the Berendsen barostat. Following the NPT equilibration, a production run was performed in the NVT (constant number of particles, volume, and temperature) ensemble at 298 K for 2 ns. This NVT phase was executed in two 1 ns stages with a time step of 1 fs.

Hydrogen bond analysis was performed on the trajectory data collected from the final 1 ns of the NVT simulation. This analysis quantified both intermolecular and intramolecular interactions, specifically examining hydrogen bond distances and the total number of hydrogen bonds formed within the systems.

**Light scattering analysis.** The optical scattering properties of both nanofibers and nanopores were calculated using an analytical Mie theory framework. The model solves Maxwell's equations, with the scattering efficiency $Qsca$ and cross-section $Csca$ derived from the continuity conditions of electromagnetic fields ^[4]^:

$$\begin{aligned} C_{sca}=\frac{1}{k^{2}}\int\int F\left( \theta,\varphi\right)sin\theta d\theta d\varphi,\#\left( 1 \right) \end{aligned}$$

The scattering coefficients $a_{n}^{\mathrm{TE}/\mathrm{TM}}$  for transverse electric (TE) and magnetic (TM) modes were computed by evaluating Bessel functions (Jn, Yn, Hn) and their derivatives at the size parameter $x=2\pi n_{m}R/\lambda$, further details on the Bessel functions (Jn, Yn, Hn) can be found on page 73 in Ref. ^[5]^.

The truncation order $N_{max}=[x+4x^{1/3}+2]$  ensured numerical convergence. The total scattering efficiency was obtained by summing over all modes ^[4, 6]^:

$$\begin{aligned} Q_{sca}= \frac{2}{x}\sum_{n=-\infty}^{\infty} \left| a_{n} \right|^{2}\#\left( 2 \right) \end{aligned}$$

The simulation was conducted over a wavelength range of 0.25 µm to 2.5 µm, covering the entire solar spectrum. The cellulose fiber diameter ranged from 0.2 µm to 30 µm, while the air pore diameter varied between 0.2 µm and 20 µm, with an incremental step size of 0.05 µm. By interpolating these data points, a contour plot was generated, illustrating the relationship between scattering efficiency and wavelength as a function of cellulose fiber diameter or air pore diameter.

**Electromagnetic scattering simulation.** The electromagnetic scattering properties were simulated using the Finite Element Method (FEM) within COMSOL Multiphysics®. The simulation was performed using the "Electromagnetic Waves, Frequency Domain" physics interface. A two-dimensional (2D) model was constructed to solve for the total field under plane wave excitation, assuming a time-harmonic convention of exp(+iωt). The geometry, including the position and dimensions of the air pores and cellulose fibers, was derived from scanning electron microscopy (SEM) images of the physical sample. These images were binarized and statistically analyzed in MATLAB, and the resulting structure was subsequently generated in the COMSOL environment via LiveLink™ for MATLAB, as depicted in Fig. S11. The simulation domain was a 15 µm × 60 µm rectangular area. Periodic boundary conditions were applied to the top and bottom boundaries. The left and right boundaries were terminated with ports and Perfectly Matched Layers (PMLs). The PML thickness and mesh refinement were carefully optimized to suppress spurious reflections and ensure the accuracy of the far-field calculations, with the mesh resolution maintained at a minimum of five elements per wavelength. The material dispersion was defined by a wavelength-dependent complex dielectric constant, ε(λ). The refractive index of the cellulose fibers was set to 1.45 ^[7]^, while the refractive index of the air pores was set to 1.0. The structure was illuminated by a normally incident plane wave.

**Ground surface temperature in China.** The ground surface temperature is analyzed using a one-dimensional unsteady heat conduction equation. The governing equation is provided in Eqs. (3) and (4), with boundary conditions specified in Eq. (5). The ground is assumed to be adiabatic at infinity.

$$\begin{aligned} \rho c_{p}\frac{\partial T}{\partial t}=\frac{\partial}{\partial x}\left[ \lambda\frac{\partial T}{\partial x} \right],\#\left( 3 \right) \end{aligned}$$

$$\begin{aligned} \frac{T\left( x,t+1 \right)-T\left( x,t \right)}{\Delta t}=a\frac{T\left( x+1,t \right)-2T\left( x,t \right)+T\left( x-1,t \right)}{\Delta x^{2}},\#\left( 4 \right) \end{aligned}$$

$$\begin{aligned} x=0,\frac{\partial T}{\partial x}=R_{n}-Q_{H}-Q_{\mathrm{LE}},x=\infty,\frac{\partial T}{\partial x}=0,\#\left( 5 \right) \end{aligned}$$

where c is the specific heat, p is the density and k is the thermal conductivity. R is total radiation and $Q_{H}+Q_{\mathrm{LE}}$ are conductive and convective and evaporative heat transfer, For governing equation, x = 0 is the layer of solid in contact with the ambient air.

For radiation ^[8]^, including solar radiation and longwave radiation,

$$\begin{aligned} R=Q_{sun}+{L-q}_{emi},\#\left( 6 \right) \end{aligned}$$

where $Q_{sun}$ is absorbed by the solar radiation, including direct and diffuse solar radiation, *L* is Radiation from Atmosphere, where $q_{\mathrm{emi}}$ is radiation emitted by the film.

Solar radiation is calculated as follows:

$$\begin{aligned} q_{sun}=\frac{S}{1000}\int_{0}^{\infty} I_{AM1.5}\left( \lambda\right)\varepsilon_{RC}\left( \lambda\right)d\lambda,\#\left( 7 \right) \end{aligned}$$

$$\begin{aligned} {\overset{¯}{a}}_{RC}=1-\frac{\int_{0}^{\infty} I_{AM1.5}(\lambda)\varepsilon_{RC}(\lambda)d\lambda}{1000},\#\left( 8 \right) \end{aligned}$$

Longwave radiation is calculated as follows:

$$\begin{aligned} q\left( T \right)=\int d\Omega cos \theta\int_{0}^{\infty} d\lambda I_{BB}\left( T,\lambda\right)\varepsilon\left( \lambda,\theta\right),\#\left( 9 \right) \end{aligned}$$

where *Ω* is a solid angle, *θ* denotes the angle between the direction of the solid angle and the normal direction of the surface, *ε*(*λ*, *θ*) is the emissivity of the object at a wavelength *λ* and angle *θ*, and *I*_BB_(*T*, *λ*) is the spectral irradiance of a blackbody.

Heat load on the cooler due to the conductive and convective heat exchange with the environment is calculated as follows,

$$\begin{aligned} q_{c}=h\left( T_{\mathrm{amb}}-T \right),\#\left( 10 \right) \end{aligned}$$

where *h* is a combined non-radiative heat transfer coefficient that describes such conductive and convective heat exchange, $T_{\mathrm{amb}}$ is the temperature of ambient, and $T$ is the temperature of surface.

**Soil water evaporation simulation in China.** The model is referred to Li et al.’s work ^[9]^. The diffusion of the vapor above the soil can be expressed by Fick’s law ^[10]^:

$$\begin{aligned} J=-D\nabla C,\#\left( 11 \right) \end{aligned}$$

where *J* is the diffusion flux of vapor, *D* is the diffusion coefficient of vapor in air, and *C* is the molar concentration. The molar concentration can be further expressed with absolute humidity (AH):

$$\begin{aligned} C=\frac{AH}{M_{H_{2}O}},\#\left( 12 \right) \end{aligned}$$

where $M_{H_{2}O}$ is the molar mass of water. The absolute humidity could be expressed by follower equation:

$$\begin{aligned} AH=\frac{RH\times P_{s}}{P_{w}\times T},\#\left( 13 \right) \end{aligned}$$

where $RH$ is the relative humidity, $P_{s}$ is saturation vapor pressure, $P_{w}$ is the specific gas constant for water vapor, and T is the temperature. According to the equation proposed by Wagner and Pruss ^[11]^, the saturation vapor pressure of water at a given temperature T can be written as:

$$\begin{aligned} P_{s}=P_{c}\exp\left( \frac{T_{c}}{T}\left( a_{1}\tau+a_{2}\tau^{1.5}+a_{3}\tau^{3}+a_{4}\tau^{3.5}+a_{5}\tau^{4}+a_{6}\tau^{7.5} \right) \right),\#\left( 14 \right) \end{aligned}$$

where $P_{c}$ is critical pressure of water, $T_{c}$ is critical temperature of water, $a_{1}, a_{2},\ldots,a_{6}$ is empirical constants, and $\tau=1-\frac{T}{T_{c}}$. Thus, molar concentration could be expressed by temperature and relative humidity. The amount of water loss by soil over a time period can be expressed as:

$$\begin{aligned} m_{loss}=J\times S_{soil}\times M_{H_{2}O}\times t,\#\left( 15 \right) \end{aligned}$$

where $S_{soil}$ is the area of soil surface and $t$ is the time. Using the aforementioned diffusion model, the fluxes of vapor diffusion above the bare soil and the soil with commercial and SRCMes were calculated.

The local weather data of different cities and the corresponding surface temperatures acquired from the heat transfer model were used to obtain preliminary soil mositure evaporation from bare soil (*m_loss_soil_*), soil under commercial mulch (*m_loss_com_*), and cooling film (*m_loss_cool_*) using the proposed model. However, since these values were calculated based on a fixed assumed amount of soil water, they may differ from the actual value. Therefore, the soil evaporation ratios under commercial mulch (*k_com_*) and SRCM (*k_cool_*) were calculated using equation (22). Eventually, the soil moisture evaporation rate under commercial mulch (*Eb_com_*) and SRCM (*Eb_cool_*) were predicted by using equation (23), based on the average soil evaporation rate (*Eb_soil_*) from 2024 obtained from satellite observation ^[12]^.

$$\begin{aligned} k_{com / cool}=\frac{m_{loss\_com/cool}}{m_{loss_{\_}soil}},\#\left( 16 \right) \end{aligned}$$

$$\begin{aligned} {Eb}_{com / cool}={Eb}_{soil}*k_{com / cool},\#\left( 17 \right) \end{aligned}$$

**Weather data.** The input weather parameters were obtained from the fifth generation of the European Centre for Medium-Range Weather Forecasts (ECMWF) atmospheric re-analysis for global climate ^[12]^ (ERA5). Produced by the Copernicus Climate Change Service (C3S) at ECMWF, ERA5 provides data with a spatial resolution of 0.25°. The simulation spanned a period of 8760 h, starting at 08:00 on January 1, 2023 (UTC + 8). Supplementary **Table 5** provides an overview of the data sources used in the simulation, among which the reanalysis data are accumulated over a 1-h period and are expressed in joules per square meter (J·m^−2^). To convert these values to watts per square meter (W·m^−2^), the accumulated values must be divided by the accumulation period in seconds.

**Greenhouse Energy Saving.** In addition to purely passive cooling methods, SRCM can also be integrated with other active cooling techniques for greenhouses. One potential application scenario is the intelligent glass greenhouse. While temperature can be controlled using various active cooling devices in such greenhouses, the associated energy consumption and carbon emissions can be significant concerns. Therefore, we developed a greenhouse model using EnergyPlus to evaluate the energy-saving and carbon emission reduction potential of the proposed SRCM in conjunction with active temperature control.

The EnergyPlus simulation process employs SketchUp software to develop a 3D model of the target greenhouse^[13, 14]^. The Venlo-type glass greenhouse in the study is a single-span structure consisting of three ridges. The north-south length is 10.8 m, with a single ridge span of 3.6 m. The east-west length is 50.4 m, divided into 14 sections, each 3.6 m long ^[15]^. The height excluding the ridge is 4.5 m, while the height including the ridge is 5.5 m (Fig. S22). In the EnergyPlus software, the glass exterior walls of the greenhouse are conceptualized as equivalent to windows in conventional buildings. This analogy is based on their transparency properties across ultraviolet (UV), visible, and mid-infrared wavelengths. Similarly, the soil and mulch components are analogously represented as the floors within building structures ^[7]^.

We considered three representative soil cover methods in the simulation: bare soil, conventional commercial mulch, and SRCM. The corresponding optical parameters are listed in Table S2. We assumed that a heat pump is used for temperature control within the greenhouse, maintaining a temperature not exceeding 30 °C and not falling below 15 °C. The coefficient of performance (COP) of the heat pump is assumed to be 3, with a power rating of 50 kW. Carbon emissions are calculated based on energy consumption.

**Carbon Emissions Saving.** The annual carbon emissions ($R_{\mathrm{CO}_{2}}$) attributable to a building's operational energy usage are determined from its annual electricity consumption for space heating and cooling. The calculation is governed by the following equations, which link energy consumption to emissions via region-specific carbon emission factors ^[16]^. To compute carbon emissions based on these equations, it is essential to determine $EF_{\mathrm{grid},\mathrm{OM}}$ and $EF_{\mathrm{grid},\mathrm{BM}}$, representing the operating margin emission factor and the build margin emission factor, respectively. The Chinese government annually releases the values of $EF_{\mathrm{grid},\mathrm{OM}}$ and $EF_{\mathrm{grid},\mathrm{BM}}$ for various power grids. In this study, the most recent CO₂ emission factors for different power grids, as published for 2023, have been utilized. Details regarding these factors can be found in Ref.^[17]^.

$$\begin{aligned} R_{\mathrm{CO}_{2}}=Q_{e}\times EF_{\mathrm{grid},\mathrm{CM}},\#\left( 18 \right) \end{aligned}$$

$$\begin{aligned} EF_{\mathrm{grid},\mathrm{CM}}=EF_{\mathrm{grid},\mathrm{OM}}\times F_{\mathrm{OM}}+EF_{\mathrm{grid},\mathrm{BM}}\times F_{\mathrm{BM}},\#\left( 19 \right) \end{aligned}$$

where $R_{\mathrm{CO}_{2}}$ represents the annual carbon dioxide emissions, quantified in kg·m⁻²·yr⁻¹, while $Q_{e}$ represents the total annual electricity consumption attributed to space cooling and heating. The parameter $EF_{\mathrm{grid},\mathrm{CM}}$ refers to the combined margin (CM) emission factor for grid-connected power systems, expressed in gCO₂·kWh⁻¹.The factor $EF_{\mathrm{grid},\mathrm{OM}}$ corresponds to the operating margin (OM) emission factor, and its associated weight, $F_{\mathrm{OM}}$, is assigned a standardized value of 0.75. Conversely, $EF_{\mathrm{grid},\mathrm{BM}}$ signifies the build margin (BM) emission factor, with its corresponding weight factor, $F_{\mathrm{BM}}$, set to 0.25 ^[18]^.

**Fig. S1.** Preparation process of the SRCM derived from waste agricultural residues (dried corn leaves).

**Fig. S2.** Process photographs for extracting high-purity cellulose from waste corn leaves. (**a**) Corn leaf raw materials soaked in the initial treatment solution. (**b**) After alkaline pretreatment, the dissolution of lignin and hemicellulose turns the solution deep yellow. (**c**) The pretreated fibers are transferred to a hydrogen peroxide solution for final oxidative bleaching to completely remove residual impurities.

**Fig. S2** visually illustrates the key physical transformation process during the efficient conversion of waste corn leaves into high-purity cellulose raw materials using a two-step “alkaline treatment–hydrogen peroxide oxidation” method. In the first step, alkaline pretreatment, corn leaf raw materials are immersed in an alkaline solution (Fig. S2a). As the reaction progresses, most of the lignin and hemicellulose are degraded and dissolved, turning the treatment solution into a deep yellow-brown color (Fig. S2b), which provides direct evidence of the effectiveness of the initial delignification process. To further purify the material, the fibers are transferred to a fresh hydrogen peroxide solution for the second step—oxidative bleaching—after completing the alkaline treatment and subsequent washing (Fig. S2c). During this final stage, the residual-colored impurities are completely removed, resulting in a color change of the fibers from yellow-brown to bright white, thereby yielding high-purity cellulose. This series of visual transformations clearly demonstrates the success of this two-step method in efficiently separating and removing lignin and hemicellulose, providing an ideal purified biomass material for the construction of advanced functional materials.

**Fig. S3.** X-ray diffraction (XRD) patterns of maize leaves, CMF, CNF, and SRCM.

**Fig. S4.** Spatial distribution profiles of hydrogen bond lengths characterizing OH-OH and OH-COOH interactions. **(a)** Intramolecular hydrogen bonds. **(b)** Intermolecular hydrogen bonds. **(c)** All hydrogen bonds (total).

**Fig. S5.** Comparison of intramolecular and intermolecular hydrogen bond numbers during the simulation.

**Fig. S6.** Comparison of microstructure and porosity of SRCMs at different CNF concentrations.

Fig. S6 reveals the influence of cellulose nanofiber (CNF) concentration on the microstructure and porosity of the SRCMs. SEM images show that the SRC without CNFs exhibits a highly porous and open fibrous network structure. As the CNF concentration increases from 2.5% to 10%, the interstitial voids within the fiber matrix are progressively filled, resulting in a denser and more compact surface morphology. Corroborating this structural change, the SRCM's porosity systematically decreases from approximately 28% (at 0% CNF) to 12% (at 10% CNF), confirming that the addition of CNFs enhances film compactness by reducing void space.

**Fig. S7.** Schematic diagram of the SRCM’s stress-strain performance testing device.

**Fig. S8**. Effect of the cooler’s optical properties on the passive radiative cooling (PRC) performance. The cooling power of the sky‐facing surface is evaluated under an irradiance of 1,000 Wm⁻² and an operating temperature of 35 °C. To examine the dependence of cooling power on *R_solar_*, *ε_AT_* is set to 0.9, which is close to the typical values for soil and most polymer plastic mulches (**a**). To examine the dependence on *ε_AW_*, the solar reflectance is set to 0.9 (**b**). The results indicate that, even under skies with different degrees of transparency, the cooling power increases with increasing reflectance and emissivity.

**Fig. S9.** Scattering electric field intensity distributions when the air pore diameter is 0.5 μm, and the incident light wavelengths are (**a**) 0.3 μm, (**b**) 0.5 μm, and (**c**) 2.5 μm. The simulations clearly demonstrate that when the pore diameter is comparable to the incident wavelength, extremely strong scattering is generated. In contrast, when the incident wavelength is shorter or longer than the pore diameter, the scattering efficiency decreases significantly.

**Fig. S10.** Scattering electric field intensity distributions when the cellulose fiber diameter is 0.5 μm, and the incident light wavelengths are (**a**) 0.3 μm, (**b**) 0.5 μm, and (**c**) 2.5 μm. The simulations clearly demonstrate that when the fiber diameter is comparable to the incident wavelength, extremely strong scattering is generated. In contrast, when the incident wavelength is shorter or longer than the pore diameter, the scattering efficiency decreases significantly.

**Fig. S11.** Summary of the supplementary idealized sensitivity study for representative submicron pores in cellulose (D_eq_ = 0.50 μm). (**a**) Shape sensitivity of the normalized side-scattering response for different aspect ratios (AR = 1–3). (**b**) Summary view of the spacing/coupling sensitivity, expressed as the ratio between the two-feature response and twice the isolated-feature response for different normalized inter-feature gaps.

**Fig. S11** provides a supplementary idealized sensitivity study to further assess the robustness of the size-resolved scattering interpretation. **Fig. S11a-b** shows that varying the feature aspect ratio from 1 to 3 changes the scattering magnitude only moderately while preserving the overall wavelength dependence, indicating that the principal size–wavelength matching trend is not restricted to a perfect circular geometry.

**Fig. S12.** Detailed inter-feature spacing sensitivity analysis for representative submicron pores in cellulose (D_eq_ = 0.50 μm). (**a**) Normalized two-feature scattering spectra for different gap-to-equivalent-diameter ratios (gap/D_eq_ = 0.1, 0.5, 1.0, and 2.0). (**b**) Coupling sensitivity ratio, defined as the ratio between the two-feature response and twice the isolated-feature response. (**c**) Angular scattering patterns of the two-feature configurations at λ = 0.50 μm. (**d**) Zoomed binary geometries illustrating the smallest and largest normalized gaps used in the idealized calculations.

**Fig. S12a-b** further show that reducing the spacing between neighboring features introduces measurable coupling effects, leading to wavelength-dependent enhancement or suppression of scattering relative to the isolated-feature reference. At the same time, **Fig. S12c** demonstrates that local coupling mainly redistributes the scattered intensity with angle. Taken together, these results indicate that feature shape anisotropy and local inter-feature interactions mainly introduce quantitative modifications to scattering magnitude and angular distribution, whereas the qualitative broadband scattering mechanism remains governed primarily by the multiscale feature sizes. This supports the use of the idealized Mie-type calculations as a first-order size-resolved interpretation, while the collective electromagnetic response of the real dense morphology is more rigorously described by the SEM-derived full-wave simulations (see **Fig. S13–S14** for details).

**Fig. S13.** (**a**) SEM image and (**b**) binary image a cross-section view of the SRCM for electric field distribution in Fig. 3h.

**Fig. S14.** Electromagnetic simulations of electric field distribution in the SRCM under light illumination at wavelengths of 0.4, 0.5, 0.6, 1 and 1.3 μm.

**Fig. S15.** SRCM's functional group spectrum.

**Fig. S16.** Representative photographs of commercially available agricultural mulches used for comparison: (a) commercial reflective mulch (CRM), (b) black mulch (BM), (c) translucent white mulch (WM), and (d) commercial biodegradable mulch (CBM).

**Fig. S17.** Optical properties of the SRCM as a function of the thickness. The reflectivity increases at the solar wavelengths as the thickness increases.

**Fig. S18.** The solar reflectivity of the SRCMs was observed to vary with the concentration of CNFs. The SRCMs with CNF concentrations of 0%, 2.5%, 5%, 7.5%, and 10% exhibited reflectance of 93.83%, 93.61%, 93.31%, 92.50%, and 91.63%, respectively.

**Fig. S19.** Contact angle images of SRCM. (a) Before hydrophobic treatment: the water droplet was completely absorbed within ~1 s owing to the superhydrophilic nature of the all-cellulose network, yielding an effective contact angle of ~0°. (b) After hydrophobic treatment with nano-silica: a stable contact angle of ~149.2° was achieved.

Isopropanol was thoroughly mixed with nano-silica particles, sealed, and stirred for 1 hour before being evenly applied to the surface of the SRCM. Subsequently, the mulch was dried at 50 °C, during which isopropanol evaporated rapidly, leaving behind a uniform nano-silica layer. Nano-silica is colorless and transparent, having minimal impact on the optical properties of the SRCM. Moreover, silica, being a primary component of sand and stones in soil, is used in small amounts and does not cause environmental pollution even after degradation in soil. The contact angle measurement was conducted by gently placing a 10 μL droplet of deionized water on a horizontal surface, followed by capturing images with a high-speed camera (Phantom, Micro C110). Due to the abundance of surface hydroxyl groups and the porous fibrous architecture, the pristine SRCM exhibited superhydrophilic behavior; the deposited water droplet was rapidly and completely absorbed, resulting in a steady-state contact angle of approximately 0° (**Fig. S19a**), whereas the hydrophobically treated SRCM exhibited a high contact angle of 149.2° (**Fig. S19b**).

**Fig. S20.** Environmental durability of the hydrophobic coating on SRCM, evaluated by (a) a abrasion test, (b) immersion in an HCl solution (pH = 2), and (c) accelerated UV-aging tests. Data points represent mean values of five independent measurements (n = 5); error bars denote standard deviation.

**Fig. S21.** Solar reflectivity of the SRCM before and after the hydrophobic treatment. The solar reflectivity of the hydrophobically treated SRCM decreased by only 1% compared to the untreated SRCM.

**Fig. S22.** Water immersion stability evaluation of the hydrophobically treated SRCM. (a) Photographs of the SRCM floating on water and under weight-assisted immersion. (b) Visible photographs of the SRCM before (0 h) and after (24 h) immersion. (c) Solar reflectance spectra of the SRCM after immersion for 0, 3, 6, 12, and 24 h.

**Fig. S23.** Solar reflectivity of the SRC before and after UV exposure in a UV aging test machine. The results showed very little degradation of the SRC after continuous UV exposure for 2 weeks, demonstrating a high UV resistance for outdoor applications.

**Fig. S24.** Schematic of the indoor experimental setup for testing temperature response (left) and water evaporation loss (right). On the left, the soil was placed in a thermally insulated box, and type‑K thermocouples were embedded at predefined depths to monitor the soil temperature beneath the sample in real time. On the right, wet soil was placed on an electronic balance, and the mass loss over time was recorded to quantify the water evaporation rate. A small hole in the mulch allowed water vapor to escape while maintaining the coverage conditions during irradiation.

**Fig. S25**. Indoor Photothermal and Moisture Evaporation Testing Setup. The left image represents the setup without mulch, while the right image shows the setup with mulch.

**Fig. S26**. Thermal imaging of the soil surface covered with different mulches after 5 minutes of exposure to varying light intensities (ambient temperature: 20 °C).

**Fig. S27.** Soil surface and deep layer temperature profiles of different mulches under simulated solar irradiance.

**Fig. S28.** Outdoor soil-based cooling testing setup for different mulch treatments.

**Fig. S29.** Calculated net cooling power of SRCM as a function of the surface-to-ambient temperature difference under different heat transfer coefficients.

To further quantify the influence of fluctuating outdoor conditions on radiative cooling performance, the net cooling power of SRCM was theoretically evaluated as a function of the surface-to-ambient temperature difference $\left( T_{s} - T_{a} \right)$under different effective heat transfer coefficients. As shown in **Fig. S25**, the calculated cooling power varies systematically with both $\left( T_{s} - T_{a} \right)$and the external heat transfer coefficient, indicating that stronger non-radiative heat exchange leads to a steeper dependence of cooling power on temperature difference. At $T_{s}=T_{a}$, the predicted net cooling power is approximately 100 W/m^2^. These results provide a quantitative framework for understanding the robustness of SRCM cooling behavior under variable field environments.

**Fig. S30.** Measurements of soil surface temperature and humidity covered with different mulches over 48 hours in Hong Kong, alongside corresponding ambient air temperature and solar irradiance.

**Fig. S31.** Measurements of soil deep-layer temperature covered with different mulches over 48 hours in Hong Kong.

**Fig. S32.** Map of the seven major geographical divisions of China.

**Fig. S33.** Surface temperatures of the (**a**) bare soil, (**b**) commercial reflective mulch, and (**c**) SRCM at hottest time during summer in China.

**Fig. S34.** Monthly soil water evaporation rates of the bare soil (**a**), commercial reflective mulch (**b**), and SRCM (**c**) during summer in China.

**Fig. S35.** Outdoor field plant growth experiment setup.

**Fig. S36.** Photos depicting growth trends of plants after germination under different mulches.

**Fig. S37.** Composting experiment setup.

Air passes through the soda lime assembly in (**a**), where moisture and carbon dioxide are absorbed, before entering the degradation chamber in (**b**) for the degradation reaction. The carbon dioxide generated during the reaction is collected in the absorption liquid assembly in (**c**), and the data on carbon dioxide release is used to calculate the biodegradation rate.

**Fig. S38**. (**a**) Biosafety experimental setup, (**b**) Earthworm survival in the experimental group (with SRCM degradation products) after 14 days. (**c**) Earthworm survival in the control group (without SRCM degradation products) after 14 days.

**Fig. S39.** Greenhouse model used in EnergyPlus simulation. The Venlo-type glass greenhouse used in the EnergyPlus simulation is a single-span structure with three ridges. The greenhouse has a north-south length of 10.8 m, with each ridge spanning 3.6 m. Its east-west length is 50.4 m, consisting of 14 sections, each 3.6 m long. The height excluding the ridge is 4.5 m, while the total height including the ridge is 5.5 m.

**Fig. S40.** Annual cooling energy consumption of a Venlo-type greenhouse under bare soil, CRM, and SRCM in selected cities.

**Fig. S41.** Map of annual energy savings across China achieved with SRCM relative to bare soil.

**Supplementary Table 1.** Comparison of current PRC technologies versus the proposed SRCM in agricultural applicability.

| **Technology Category** | **Material Availability** | **Preparation & Scalability** | **Degradability & Sustainability** | **Radiative Cooling Performance** | **Primary Limitation for Agriculture** |
| --- | --- | --- | --- | --- | --- |
| **Photonic Micro/ Nanostructures-based PRC** ^[19]^ | Synthetic materials generally require high purity. | High difficulty.  Involves complex lithography/etching, high cost, and limited scalability. | **Non-biodegradable.** Potential source of microplastic pollution. | Rsolar% ~ 93 %.  E% ~ 92 %.  Sub-ambient cooling  ~ 3 °C.  Soil cooling ~ 12.5 °C | High cost and high sensitivity to field contamination. |
| **Inorganic-filled or Porous Polymers-based PRC** ^[20, 21]^ | Petroleum-derived polymers combined with inorganic fillers. | Medium difficulty.  Energy-intensive processing with high filler loading. | **Poor.** Generates persistent plastic waste; difficult to remove from soil and to recycle. | Rsolar% ~ 96 %.  E% ~ 97 %.  Sub-ambient cooling  ~ 6 °C | Environmental pollution and lack of circularity. |
| **Synthetic Biopolymers-based PRC** ^[22, 23]^ | Biobased polymers (e.g., PLA, EA, CA) typically require dedicated extraction or synthesis. | Medium difficulty.  Relies on chemical solvents and/or electrospinning; generally high energy consumption. | Biodegradable**.**  Residues may inhibit crop growth; often slow degradation. | Rsolar% ~ 92 %, 96 %.  E% ~ 95 %, 85 %.  Sub-ambient cooling  ~ 5.7 °C | Solvent toxicity and poor compatibility with crop rotation. |
| **SRCM**  **(This Work)** | **Agricultural residues (maize leaves); abundant, locally available, and effectively zero‑cost.** | **Low difficulty.**  **Scalable, aqueous, hydrogen‑bond-driven self-assembly without external energy input.** | **Biodegradable.**  **with high recyclability and biosafety.** | **Rsolar% ~ 93.3 %.**  **E% ~ 92.6 %.**  **Sub-ambient cooling ~ 4.1 °C.**  **Soil cooling ~ 18 °C** | **None identified; specifically tailored for high-cooling performance, sustainable agricultural deployment.** |

**Supplementary Table 2.** Comparison of current degradable cellulose-based PRCs versus the SRCM in agricultural applicability.

| **Ref.** | **Material form** | **Cellulose source** | **Key fabrication** | **Solvent of molding process** | **Additive** | **PRC**  **performance** | **Bio-**  **degradability** | **Agricultural compatibility** |
| --- | --- | --- | --- | --- | --- | --- | --- | --- |
| [24] | Cooling wood | Balsa wood | Vacuum Immersion | Water, ethanol | Mica, TiO_2_ | R% ~ 95.8 %  E% ~ 95 %  ΔT_sub-amb_ ~ 7.5 °C | Not reported | Rigid block,  Residual inorganic matter after degradation. |
| [25] | Colored  cooling wood | Wood,  CNC | Freeze-drying; hot pressing; oven drying | Water, | PVA,  PDMS | R% ~ 95 %  E% ~ 93 %  ΔT_sub-amb_ ~ 4.3 °C | Not reported | Rigid block. |
| [26] | Aerogel | Rice straw | Freeze-drying, chemical vapor deposition | DMF, sulfamic acid, urea. | SiO_2_ | R% ~ 97.6 %  E% ~ 96.6 %  ΔT_sub-amb_ ~ 12.5 °C | 100% in 45 days | Difficult to scale,  High cost and solvent risks. |
| [27] | Aerogel | Wood | Freeze-drying, hydrothermal reaction | Aqueous ammonia,  tetraethyl orthosilicate. | SiO_2_ | R% ~ 96.43 %  E% ~ 97.49 %  ΔT_sub-amb_ ~ 15.6 °C | Not reported | Difficult to scale,  Solvent risks. |
| [28] | Fabric | Basswood | Wet spinning, freeze-drying | Alkaline urea, epichlorohydrin,  phytic acid coagulation | SiO_2_ | R% ~ 96.43 %  E% ~ 97.49 %  ΔT_sub-amb_ ~ 15.6 °C | Not reported | High fabrication cost and solvent risks. |
| [29] | Fabric | Cellulose acetate | Electrospinning | HFIP,  DMF,  acetone | SiO_2_ | R% ~ 97.13 %  E% ~ 97.70 %  ΔT_sub-amb_ ~ 14.3 °C | Not reported | High fabrication cost and solvent risks. |
| [30] | Fabric | Cellulose acetate | Electrospinning | Trichloromethane,  DMF,  acetone | MOF (ZIF-8) | R% ~ 95.7 %  E% ~ 94 %  ΔT_sub-amb_ ~ 5.7 °C | Not reported | High fabrication cost and solvent risks. |
| [31] | Coating | Ethyl cellulose | Phase separation | Water, DMF | None | R% ~ 97 %  E% ~ 92.1 %  ΔT_sub-amb_ ~ 9 °C | Not reported | High raw material costs; solvent risks, |
| [32] | Film | Bamboo | Vacuum filtration, hot pressing | Water | h-BN,  SiO_2_, PVA | R% ~ 98.1 %  E% ~ 93.2 %  ΔT_sub-amb_ ~ 8.9 °C | Not reported | High fabrication cost,  Residual inorganic matter after degradation. |
| [9] | Film | Ethyl cellulose | Phase separation | Water, ethanol | None | R% ~ 97 %  E% ~ 93 %  ΔT_sub-amb_ ~ 6 °C | 4.5% in 30 days | High raw material costs; Insufficient degradation rates. |
| This work | Film | Maize | Natural drying | Water | None | R% ~ 93.3 %  E% ~ 92.6 %  ΔT_sub-amb_ ~ 4.1 °C | 100% in 3 months | Scalable, locally sourced materials, low environmental footprint, moderate degradation rate. |

PRC means passive radiative cooling; CNC means cellulose nanocrystal; R% means reflectance; E% means emissivity; ΔT_sub-amb_ means sub-ambient cooling temperature.

**Supplementary Table 3.** Performance comparison for SRCM, CCM, CRM, and CEM.

|  | **SRCM** | **Commercial Conventional Mulch** | **Commercial Reflective Mulch** | **Commercial Biodegradable Mulch** |
| --- | --- | --- | --- | --- |
| Cooling ^a^ | 15-20℃  +++++ | 62-67℃  + | 40-45℃  ++ | 40-45℃  ++ |
| Degradability | 3 months  +++++ | 200 years  + | 200 years  + | 3 months  +++++ |
| Recyclability | Stirring and re-casting  +++++ | Mechanical, Incineration， Melt Pelletizing  ++ | Mechanical, Incineration， Melt Pelletizing  ++ | Stirring, mixing with chemical binders, and re-casting.  ++++ |
| Water Saving ^b^ | - 65.06 g  +++++ | -126.15 g  +++ | -104.24 g  ++++ | -106.70 g  ++++ |
| Sustainability | Yes  +++++ | No  + | No  + | Yes  ++++ |
| Strength | 10.1-17.8 MPa  +++++ | 2-4 MPa  ++ | 3-5 MPa  +++ | 10-12 MPa  ++++ |

^a^ Measured as the undersurface temperature of the mulches under identical solar irradiation conditions; lower temperatures indicate superior cooling performance.

^b^ Calculated based on water loss per square meter per hour under identical test conditions; lower water loss indicates better water-saving efficiency.

**Supplementary Table 4.** Air permeability of different mulches.

| **Sample** | **Air permeability / [cm^3^/(m^2^·d·Pa)]** |
| --- | --- |
| WM | 1.277 × 10^-3^ |
| BM | 4.655 × 10^-3^ |
| CRM | 1.928 × 10^-3^ |
| CBM | 2.294 × 10^5^ |
| SRCM | 1.586 × 10^4^ |

**Supplementary Table 5.** Input parameters from ERA5.

| **Symbols** | **Full name** | **Units** |
| --- | --- | --- |
| u10 | 10mU wind component | m.s^-1^ |
| v10 | 10mV wind component | m.s^-1^ |
| fdir | Total sky direct solar radiation at a surface | J·m^-2^ |
| ssrd | Surface short-wave radiation downwards | J·m^-2^ |
| strd | Surface long-wave radiation downwards | J·m^-2^ |
| t2m | 2 m temperature | K |

**Supplementary Table 6**. Optical properties of materials employed in EnergyPlus software.

| **0.25-2.5μm** | **Bare Soil** | **Commercial Reflective** | **SRC** |
| --- | --- | --- | --- |
| Transmission | 0 | 0 | 0 |
| Emissivity | 0.81 | 0.49 | 0.075 |
| Reflectance | 0.19 | 0.51 | 0.925 |
| **8-13μm** | **Bare Soil** | **Commercial Reflective** | **SRC** |
| Transmission | 0 | 0 | 0 |
| Emissivity | 0.98 | 0.94 | 0.93 |
| Reflectance | 0.02 | 0.06 | 0.07 |

References

[1].	Zhu, M., et al., Highly Anisotropic, Highly Transparent Wood Composites, 2016, Advanced Materials (Deerfield Beach, Fla.), **28**(26): p. 5181-5187

[2].	Hop, T.T.T., et al., A comprehensive study on preparation of nanocellulose from bleached wood pulps by TEMPO-mediated oxidation, 2022, Results in Chemistry, **4**: p. 100540

[3].	Song, J., et al., Durable radiative cooling against environmental aging, 2022, Nature Communications, **13**(1): p. 4805

[4].	Thovsen, K.B., Evaluation of Mie scatter approximation formulas for the scattering of infrared light at biological cells, 2013, Norwegian University of Life Sciences,

[5].	Seyedheydari, F., et al. *Electromagnetic Response and Optical Properties of Spherical CuSbS 2 Nanoparticles*. in *2021 Photonics & Electromagnetics Research Symposium (PIERS)*. 2021. IEEE.

[6].	Pettersson, R., Frequency dependence of scattering from the cold-temperate transition surface in a polythermal glacier, 2005, Radio Science, **40**(3): p. 1-7

[7].	Tian, Y., et al., Passive cooling of greenhouses in extreme climates through spectral control film, 2025, Nexus, **2**(1)

[8].	Fan, S. and W. Li, Photonics and thermodynamics concepts in radiative cooling, 2022, Nature Photonics, **16**(3): p. 182-190

[9].	Lin, C., et al., Alleviating heat stress in cultivated plants with a radiative cooling and moisturizing film, 2024, Energy Conversion and Management, **315**: p. 118786

[10].	Conlisk, A.T., *Essentials of micro-and nanofluidics: with applications to the biological and chemical sciences*. 2012: Cambridge University Press.

[11].	Wagner, W. and A. Pruß, The IAPWS formulation 1995 for the thermodynamic properties of ordinary water substance for general and scientific use, 2002, Journal of Physical and Chemical Reference Data, **31**(2): p. 387-535

[12].	Miralles, D.G., et al., GLEAM4: global land evaporation and soil moisture dataset at 0.1 resolution from 1980 to near present, 2025, Scientific Data, **12**(1): p. 416

[13].	Li, X., et al., Integration of daytime radiative cooling and solar heating for year-round energy saving in buildings, 2020, Nature Communications, **11**(1): p. 6101

[14].	Zou, H., et al., Eliminating greenhouse heat stress with transparent radiative cooling film, 2023, Cell Reports Physical Science, **4**(8)

[15].	Jin, Y., et al., Comprehensive optimization of shading and electrical performance of roof-mounted photovoltaic system of Venlo-type greenhouse in the severe cold region, 2024, Energy, **296**: p. 131125

[16].	Zhang, Z., et al., Carbon mitigation potential afforded by rooftop photovoltaic in China, 2023, Nature Communications, **14**(1): p. 2347

[17].	NCSC, 2023 Emission reduction project China regional power grid baseline emission factors, 2024,

[18].	Segal, M.R., Machine learning benchmarks and random forest regression, 2004,

[19].	Wang, C., et al., Enhancing food production in hot climates through radiative cooling mulch: A nexus approach, 2024, Nexus, **1**(1)

[20].	Zhai, Y., et al., Scalable-manufactured randomized glass-polymer hybrid metamaterial for daytime radiative cooling, 2017, Science, **355**(6329): p. 1062-1066

[21].	Mandal, J., et al., Hierarchically porous polymer coatings for highly efficient passive daytime radiative cooling, 2018, Science, **362**(6412): p. 315-319

[22].	Qi, G., et al., Anti-aging and flexible-porous-array films for radiative cooling, 2024, Solar Energy Materials and Solar Cells, **268**: p. 112733

[23].	Chen, Z., et al., Biodegradable and Hierarchically Designed Polymer Film for Sustainable Daytime Radiative Cooling, 2023, ACS Applied Optical Materials, **2**(6): p. 928-934

[24].	Cai, C., et al., Bioinspired durable daytime radiative cooling wood: realizing outdoor longtime use, 2025, Nano Letters, **25**(11): p. 4369-4378

[25].	Sun, B., et al., Cellulose based hierarchical structural colored wood composite material for daytime radiative cooling, 2025, Carbohydrate Polymers: p. 124563

[26].	Zhao, Q., et al., Loofah-inspired cellulose-based super-white aerogel with enhanced mechanical strength for high-performance daytime radiative cooling, 2025, Composites Science and Technology: p. 111421

[27].	Long, A., et al., A Flame‐Retardant, Thermal‐Insulating Wood‐Derived Aerogel toward All‐Season Thermal Management, 2026, Advanced Functional Materials, **36**(25): p. e24221

[28].	Li, Q., et al., Eco-Friendly Skin-Wrinkle-Inspired Micro-Nano Structured Cellulose Composite Fibers for Highly Efficient Daytime Radiative Cooling, 2025, ACS Nano, **19**(39): p. 34596-34605

[29].	Zhao, M., et al., Bio-based silk fibroin-cellulose acetate Janus fabric for synergistic evaporative and radiative cooling in personal thermal management, 2026, Chemical Engineering Journal: p. 173451

[30].	Cai, C., et al., An engineered superdurable cellulosic radiative cooling–Power generation wearable metafabric, 2024, Chemical Engineering Journal, **493**: p. 152599

[31].	Liu, Y., et al., One-step-processed bilayer ethyl cellulose for full-colour sub-ambient daytime radiative cooling, 2026, Nature Energy: p. 1-11

[32].	Lin, X., et al., Strong, Recyclable, and Sustainable Radiative Cooler with Heterogeneous Interlocking Architecture for Agricultural Thermal Management, 2026, ACS Nano,
